# Metabolic changes contribute to maladaptive right ventricular hypertrophy in pulmonary hypertension beyond pressure overload: an integrative imaging and omics investigation

**DOI:** 10.1007/s00395-024-01041-5

**Published:** 2024-03-27

**Authors:** Inés García-Lunar, Inmaculada Jorge, Jorge Sáiz, Núria Solanes, Ana Paula Dantas, Juan José Rodríguez-Arias, María Ascaso, Carlos Galán-Arriola, Francisco Rafael Jiménez, Elena Sandoval, Jorge Nuche, Maria Moran-Garrido, Emilio Camafeita, Montserrat Rigol, Javier Sánchez-Gonzalez, Valentín Fuster, Jesús Vázquez, Coral Barbas, Borja Ibáñez, Daniel Pereda, Ana García-Álvarez

**Affiliations:** 1 Centro Nacional de Investigaciones Cardiovasculares Carlos III (CNIC), Madrid, Spain; 2 CIBER de Enfermedades Cardiovasculares (CIBERCV), Madrid, Spain; 3 Cardiology Department, University Hospital La Moraleja, Madrid, Spain; 4 Centre of Metabolomics and Bioanalysis (CEMBIO), Department of Chemistry and Biochemistry, Facultad de Farmacia, Universidad San Pablo-CEU, Madrid, Spain; 5 Department of Cardiology, Hospital Clínic Barcelona-IDIBAPS, Universitat de Barcelona, Villarroel 170, 08036 Barcelona, Spain; 6 Department of Cardiovascular Surgery, Hospital Clínic Barcelona, Barcelona, Spain; 7 Department of Cardiology, Hospital 12 de Octubre, Madrid, Spain; 8 Philips Healthcare Iberia, Madrid, Spain; 9 Mount Sinai Fuster Heart Hospital, Mount Sinai Hospital, New York, NY USA; 10 IIS-Fundación Jiménez Diaz University Hospital, Madrid, Spain; 11 Universitat de Barcelona, Barcelona, Spain

**Keywords:** Pulmonary hypertension, Right ventricle, Omics, Cardiac imaging

## Abstract

Right ventricular (RV) failure remains the strongest determinant of survival in pulmonary hypertension (PH). We aimed to identify relevant mechanisms, beyond pressure overload, associated with maladaptive RV hypertrophy in PH. To separate the effect of pressure overload from other potential mechanisms, we developed in pigs two experimental models of PH (M1, by pulmonary vein banding and M2, by aorto-pulmonary shunting) and compared them with a model of pure pressure overload (M3, pulmonary artery banding) and a sham-operated group. Animals were assessed at 1 and 8 months by right heart catheterization, cardiac magnetic resonance and blood sampling, and myocardial tissue was analyzed. Plasma unbiased proteomic and metabolomic data were compared among groups and integrated by an interaction network analysis. A total of 33 pigs completed follow-up (M1, *n* = 8; M2, *n* = 6; M3, *n* = 10; and M0, *n* = 9). M1 and M2 animals developed PH and reduced RV systolic function, whereas animals in M3 showed increased RV systolic pressure but maintained normal function. Significant plasma arginine and histidine deficiency and complement system activation were observed in both PH models (M1&M2), with additional alterations to taurine and purine pathways in M2. Changes in lipid metabolism were very remarkable, particularly the elevation of free fatty acids in M2. In the integrative analysis, arginine–histidine–purines deficiency, complement activation, and fatty acid accumulation were significantly associated with maladaptive RV hypertrophy. Our study integrating imaging and omics in large-animal experimental models demonstrates that, beyond pressure overload, metabolic alterations play a relevant role in RV dysfunction in PH.

**Supplementary Information:**

The online version contains supplementary material available at 10.1007/s00395-024-01041-5.

## Introduction

Right ventricular (RV) failure is the strongest determinant of survival in patients with pulmonary arterial hypertension (PAH) and independently predicts the outcome of pulmonary hypertension (PH) associated with left heart disease [8, 10]. However, the pathophysiological mechanisms leading to impaired RV function in PH are not clearly understood, and this has hampered the development of specific treatments for this indication. Clinical observations and experimental studies suggest that RV pressure overload is not the only cause of RV dysfunction in PH. For example, pediatric patients with severely increased RV pressure due to pulmonary artery (PA) stenosis, thus with normal PA pressure (PAP), develop severe RV hypertrophy but usually maintain normal RV systolic function for long periods. Similarly, some PAH patients show a progressive decline in RV function despite a successful reduction in pulmonary vascular resistance (PVR) with treatment [26]. In an experimental study in rats [2], progressive pulmonary stenosis generated by surgical PA banding was not associated with significant RV dysfunction, whereas chronic PH induced by combined exposure to hypoxia and the vascular endothelial growth factor receptor blocker SU5416 was, despite both groups of animals having similar RV systolic pressures. These collected observations suggest that RV failure in PH is dependent on other mechanisms besides RV pressure overload.

We hypothesized that a maladaptive RV response in PH would involve the activation of pathologic pathways detectable as changes in blood biomarkers. Recent advances in proteomics, metabolomics, and interaction network analysis offer a unique opportunity for the unbiased identification of biomarkers and pathways implicated in complex diseases. In addition, non-invasive imaging techniques have substantially improved RV phenotyping in PH [9] being cardiac magnetic resonance (CMR) the current gold standard technique. In the present study, we integrated omics and CMR imaging data from experimental models of chronic precapillary and postcapillary PH to identify pathophysiological mechanisms associated with RV dysfunction unrelated to pressure overload.

## Methods

### Study design and experimental models

Experimental procedures were performed in castrated male Yucatan pigs. The study was approved by the Institutional Animal Research Committee and carried out in compliance with European (2010/63/EU) and Spanish (RD 53/2013) regulations on the protection of the animals used for scientific purposes.

Animals (*n* = 46 aged 3–4 months and weighing 10–15 kg) were assigned to four surgical procedures in three different clusters of programmed surgeries (supplementary Fig. 1): a model of chronic postcapillary PH induced by surgical nonrestrictive banding of the pulmonary veins (M1); chronic PH induced by aorto-pulmonary shunting (M2); RV pressure overload induced by pulmonary artery (PA) banding, therefore without PH (M3); and a sham procedure (M0). For M1, a nonrestrictive band was inserted through a small right thoracotomy and placed around the venous confluence formed by the junction of both inferior pulmonary veins and the right superior pulmonary vein, in a variant of a previously reported model [20]. For M2, the left brachiocephalic artery was clamped, ligated, and anastomosed to the main PA in an end-to-side fashion via a small left thoracotomy [21]. In M3, the main PA was accessed via a small left thoracotomy and RV pressure overload was generated by surgical restrictive banding of the vessel with fabric tape tailored at two thirds of the vessel perimeter and secured with 2.0 silk suture thread. In M0 (sham procedure), the pulmonary vessels were accessed, exposed, and dissected via a left thoracotomy, but no banding or shunting was performed. Animals were assessed at months 1 and 8 after surgery. Follow-up included right heart catheterization and noninvasive imaging (CMR in all animals and cardiac computed tomography [CT] in models M1, M2, and M3). At the study endpoint (month 8 after surgery), animals were sacrificed by lethal injection of sodium pentobarbital (50 mg/kg intravenously), and the heart and lung parenchyma were excised for molecular biology analysis.

### Invasive hemodynamic assessment and advanced imaging studies

Right heart catheterization (RHC) and immediate CMR and CT were performed following standardized protocols that have been described previously [7, 20, 21] and are available in the Online Appendix.

### Metabolomics

Metabolomic analyses were performed in samples obtained from all animals at end of follow-up (month 8). Lipids for lipidomics analysis and polar compounds for analysis by hydrophilic interaction liquid chromatography (HILIC) were extracted from plasma using previously described protocols [15]. LC-based lipidomics and HILIC were performed with an Agilent 1290 Infinity II ultra-high performance liquid chromatography (UHPLC) system coupled either to an Agilent 6560 ion mobility quadrupole time-of-flight (IM-QTOF) mass spectrometer working in QTOF mode or to a 6545 QTOF mass spectrometer (Agilent Technologies Waldbronn, Germany). A complete description of sample preparation, the analytical parameters and procedures is available in the Online Appendix.

### Proteomics

Proteomics analysis was performed in plasma samples obtained at baseline (immediately before surgery) and 1 and 8 months after surgery from a random subset of 16 pigs (4 animals per intervention group).

Samples (100 μg protein) were on-filter digested with sequencing grade trypsin (Promega) using FASP technology (Expedeon) in a 1:40 (w/w) trypsin:protein ratio at 37 °C overnight with gentle agitation. The resulting tryptic peptides were isobarically labeled using tandem mass tags (TMT; Thermo Fisher Scientific, Bremen, Germany). A total of eight TMT experiments were performed (Supplementary Table 1). The differentially tagged peptide samples were then appropriately pooled, desalted on Waters Oasis HLB C18 cartridges, and analyzed by liquid chromatography coupled to tandem mass spectrometry (LC–MS/MS) using an Ultimate 3000 HPLC system (Thermo Fisher Scientific) coupled via a nanoelectrospray ion source (Thermo Fisher Scientific) to a Q Exactive HF mass spectrometer (Thermo Fisher Scientific).

A complete description of the sample preparation methods, the analytical procedure, and the statistical assessment of protein abundance changes is available in the Online Appendix.

### Molecular biology analysis

The expression of genes encoding proteins linked to the major pathways identified in the proteomics and metabolomics analyses was assessed by quantitative real-time PCR (qPCR) in heart and lung tissue. The plasma content of proteins implicated in the coagulation and complement cascades was measured by multiplex bead-based immunoassay. In addition, plasmatic levels of cartilage intermediate layer protein (CILP1) and N-terminal pro-brain natriuretic peptide (NT-proBNP), previously linked to maladaptive RV remodeling [12] were determined by dedicated ELISA. Detailed information is provided in the Online Appendix.

### Statistical analysis

Sample size estimation is provided in the Online Appendix. Continuous hemodynamic and imaging variables are expressed as median (interquartile range) and compared among groups by ANOVA or the Wilcoxon test according to variable distribution assessed with the Shapiro–Wilk Test. Post-hoc comparisons among M1, M2, and M3 with respect to M0 (control) were performed by ANOVA with the Dunnet correction for normally distributed data or by Mann–Whitney test (with *p* value correction) for non-normally distributed variables. Statistical analysis was performed with SPSS (version 26), and differences were considered statistically significant at a *p* value < 0.05.

For the metabolomics analyses (lipidomics and HILIC), areas were normalized with the corresponding internal standard, and missing values were imputed using a MATLAB (R2018b, MathWorks) K-nearest neighbor script. Once all the data were processed, features in the QC samples whose coefficient of variation was above 20% were discarded. Finally, for each individual feature, differences between each model (M1, M2, and M3) and the control (M0) were assessed with the Mann–Whitney *U* test (*p* ≤ 0.05), using an in-house MATLAB script with the Benjamini–Hochberg correction (α = 0.05). The percentage change for each statistically significant feature was calculated against the control model (M0), and features with a change above ± 20% were selected for annotation. More detailed information regarding features detection, selection for statistical analysis, identification of statistically significant features and final annotation can be found in the Online Appendix. Significant protein abundance changes were assessed with the Student *t*-test, with significance assigned at *p* < 0.05. Functional enrichment analysis was performed with String (https://string-db.org/) [24]; and an FDR was calculated based on the p-value obtained with the Benjamini–Hochberg procedure for each Gene Ontology category.

Pearson correlation coefficients were calculated between hemodynamic and imaging parameters and protein, lipid, and metabolite measurements. Adjustment for multiple hypothesis testing was performed by controlling for the FDR. In addition, a multivariate lineal regression analysis adjusted by weight was performed to evaluate the independent association between the main identified metabolites and RV ejection fraction. Correlation networks between hemodynamic and imaging parameters and proteins, lipids, and metabolites from M1, M2, and M0 were built with Cytoscape 3.9.1. [23].

## Results

### Cardiac adaptation characterized by hemodynamics and imaging differs among the three models of pressure overload being worse in the presence of PH

Of the 46 pigs that underwent surgery, 33 completed the follow-up, distributed as follows: M1 (*n* = 8), M2 (*n* = 6), M3 (*n* = 10), and M0 (*n* = 9). The reasons for not completing the protocol were inability to generate PH in M1 (*n* = 2), RV pressure elevation < 25 mmHg in M3 (*n* = 5), extracardiac complications (*n* = 5, 4 M0 and 1 M1 pig), and sudden death (*n* = 1 M3 pig) (Supplementary Fig. 1). Correct surgical execution of the experimental models was confirmed by CT, as follows: significant pulmonary vein stenosis in M1, significant PA stenosis in M3, and patent aorto-pulmonary shunt in M2 (Supplementary Table 3). An interim hemodynamic and CMR characterization at 1-month follow-up is summarized in Supplementary Table 4. As expected, at the final hemodynamic assessment, RV systolic pressure was significantly higher in models M1, M2, and M3 (the most severe) than in the control group (M0), whereas PAP and PVR were significantly elevated in M1 and M2, but not in M3 (Table 1).

**Table 1:** Hemodynamic characterization of animal models at end of follow-up

	M1 (*n* = 8)	M2 (*n* = 6)	M3 (*n* = 10)	M0 (*n* = 9)	*p* Value among groups
Weight, kg	58.8 (50.2–65.0)	59.5 (57.5–67.0)	67.8 (61.5–78.0)	75.0 (69.0–80.0)	0.013*γ
Oxygen saturation, %	93.5 (91.0–95.5)	93.0 (92.0–96.0)	94.5 (91.0–97.0)	95.0 (90.0–95.0)	0.939
HR, bpm	81.0 (73.5–103.0)	84.5 (79.0–104.0)	73.5 (70.0–90.0)	72.0 (66.0–78.0)	0.273
SBP, mmHg	119 (100–126)	141 (128–146)	123 (121–127)	114 (112–118)	0.006γ
MBP, mmHg	92 (84–102)	97 (93–103)	95 (89–103)	90 (83–93)	0.283
RAP, mmHg	2.0 (0.5–3.5)	1.5 (-1.0–2.0)	3.5 (2.0–5.0)	0.0 (0.0–2.0)	0.013φ
RVSP, mmHg	42.5 (37.5–44.5)	43.5 (39.0–44.0)	78.0 (55.0–95.0)	28 (26–30)	< 0.001*γφ
SPAP, mmHg	38.5 (37.5–42.5)	42.0 (36.0–48.0)	30.5 (28.0–33.5)	26.0 (23.0–27.0)	< 0.001*γ
DPAP, mmHg	27.0 (24.5–28.5)	26.0 (23.0–28.0)	23.0 (16.0–24.5)	15.0 (12.0–17.0)	< 0.001*γ
MPAP, mmHg	33.0 (31.0–35.5)	33.0 (30.0–37.0)	25.0 (22.0–26.0)	21.0 (17.0–22.0)	< 0.001*γ
LVEDP, mmHg	9.0 (5.5–10.5)	7.0 (6.0–8.0)	7.5 (7.0–10.0)	5.0 (3.0–5.0)	0.044
CO, L/min	3.8 (3.2–4.5)	3.7 (3.4–3.9)	3.8 (3.1–4.3)	3.9 (3.2–4.3)	0.859
CI (L/min/m^2^)	3.0 (2.9–3.2)	2.9 (2.5–3.0)	2.7 (2.3–2.9)	2.6 (2.3–2.7)	0.326
PVR, WU	6.3 (4.6–9.6)	6.9 (6.2–7.7)	4.6 (3.6–4.9)	3.7 (3.3–4.3)	0.001*γ
iPVR, WU*m^2^	8.5 (6.3–10.4)	8.8 (8.0–9.9)	5.8 (5.1–6.7)	6.0 (5.2–6.2)	0.003*γ

*CI* cardiac index; *CO* cardiac output; *DPAP* diastolic pulmonary arterial pressure; *HR* heart rate; *iPVR* indexed pulmonary vascular resistance; *LVEDP* left ventricular end-diastolic pressure; *MBP* mean blood pressure; *MPAP* mean pulmonary arterial pressure; *PVR* pulmonary vascular resistance; *RAP* right atrial pressure; *RVSP* right ventricular systolic pressure; *SBP* systolic blood pressure; *SPAP* systolic pulmonary arterial pressure

Data are presented as median (interquartile range)

^*^p < 0.05 for the post-hoc comparison of M1 (pulmonary vein banding) vs M0 (Control); γ *p* < 0.05 for the post-hoc comparison of M2 (aorto-pulmonary shunt) vs M0; φ *p* < 0.05 for the post-hoc comparison of M3 (pulmonary artery banding) vs M0

On CMR evaluation at end of follow-up, all three RV pressure overload models generated RV hypertrophy compared with the control group, however, RV systolic dysfunction (indicated as increased RV systolic volume and reduced RV ejection fraction) was observed only in models M1 and M2, not in M3 (Table 2). In addition, M1 showed significant RV dilatation (increased indexed RV end-diastolic volume and RV-to-LV end-diastolic volume ratio) and M2 significant LV dilatation associated with increased LV mass. T1 mapping sequences revealed an increase in ECV at the interventricular septum in M1, M2, and M3; at the inferior insertion point in M1 and M2; at the anterior insertion point in M1; and at the LV lateral wall in M2.

**Table 2:** CMR data on RV adaptation to RV pressure overload at the end of follow-up

	M1 (*n* = 8)	M2 (*n* = 6)	M3 (*n* = 10)	M0 (*n* = 9)	*p* Value
RV mass, g/m^2^	19.5 (15.7–22.4)	17.4 (16.6–17.7)	24.9 (20.3–28.9)	12.9 (12.8–14.7)	< 0.001*γφ
RV end-diastolic volume, mL/m^2^	71.5 (58.3–78.9)	63.0 (58.1–64.9)	64.8 (59.3–72.1)	55.1 (48.7–58.4)	0.042*
RV end-systolic volume, mL/m^2^	30.1 (25.7–36.0)	29.2 (23.9–32.3)	24.3 (21.7–32.5)	19.2 (15.5–23.2)	0.017*γ
RV ejection fraction, %	54.5 (50.5–59.9)	54.0 (47.0–62.0)	60.5 (56.1–67.8)	62.0 (59.0–66.5)	0.034*γ
LV mass, g/m^2^	53.8 (49.9–58.2)	67.3 (65.6–70.4)	48.2 (46.4–56.8)	47.0 (39.1–53.5)	< 0.001γ
LV end-diastolic volume, mL/m^2^	67.8 (59.1–73.1)	114.1 (91.0–134.3)	62.0 (58.9–67.6)	60.6 (53.7–68.9)	0.003γ
LV end-systolic volume, mL/m^2^	28.8 (24.2–30.7)	44.5 (36.1–47.8)	24.4 (22.6–28.9)	23.7 (19.1–29.4)	0.005γ
LV ejection fraction, %	57.0 (52.0–60.5)	61.0 (59.0–62.0)	59.5 (53.0–66.5)	62.0 (57.0–62.7)	0.446
RV to LV end-diastolic volume ratio	1.04 (0.98–1.10)	0.54 (0.48–0.64)	1.00 (0.96–1.01)	0.85 (0.83–0.94)	< 0.001*γ
RV to LV end-systolic volume ratio	1.10 (0.97–1.24)	0.60 (0.51–0.83)	1.05 (0.86–1.09)	0.84 (0.80–0.95)	< 0.001*
RV to LV mass ratio	0.38 (0.32–0.40)	0.24 (0.23–0.26)	0.55 (0.36–0.60)	0.28 (0.24–0.35)	< 0.001φ
*E*_a_/*E*_max_	0.61 (0.44–0.80)	0.40 (0.32–0.50)	0.39 (0.32–0.50)	0.37 (0.33–0.41)	0.124
PA compliance, mL/mmHg	3.89 (3.46–4.76)	4.89 (3.67–6.78)	5.74 (3.06–8.31)	5.08 (4.4–8.0)	0.569
T1 RV AIP, ms	1037 (1018–1069)	1056 (968–1100)	1078 (1039–1106)	1025 (1009–1054)	0.349
ECV RV AIP, %	30.1 (26.0–32.6)	25.0 (21.0–28.0)	24.0 (22.9–26.4)	22.0 (20.0–22.6)	0.008*
T1 RV IIP, ms	1015 (1006–1062)	1067 (994–1081)	1086 (1019–1090)	1022 (992.9–1043.5)	0.601
ECV RV IIP, %	28.3 (26.6–30.7)	28.0 (26.0–33.0)	25.0 (24.1–29.0)	22.0 (20.4–23.5)	0.010*γ
T1 IVS, ms	1026 (1007–1049)	1039 (975–1064)	1024 (973–1113)	1039 (974–1043)	0.916
ECV SIV, %	25.0 (24.0–29.2)	27.0 (24.0–27.0)	22.9 (21.4–26.5)	21.0 (19.2–21.1)	0.003*γφ
T1 LV, ms	1032 (1000–1079)	1108 (1020–1112)	1035 (986–1058)	1072 (1039–1105)	0.617
ECV LV, %	25.7 (22.2–26.5)	27.0 (25.0–29.0)	22.3 (20.6–23.2)	19.0 (19.0–20.1)	0.011γ

*AIP* anterior insertion point; *ECV* extracellular volume; *IIP* inferior insertion point; *IVP* inter-ventricular septum; *LV* left ventricle; *PA* pulmonary artery; *RV* right ventricle

Data are presented as median (interquartile range)

*p < 0.05 for the post-hoc comparison of M1 (pulmonary vein banding) vs M0 (Control); γ *p* < 0.05 for the post-hoc comparison of M2 (aorto-pulmonary shunt) vs M0; φ *p* < 0.05 for the post-hoc comparison of M3 (pulmonary artery banding) vs M0

### PH models show marked differences from the isolated RV pressure overload model in amino-acid and lipid metabolism

**Fig. 1:**
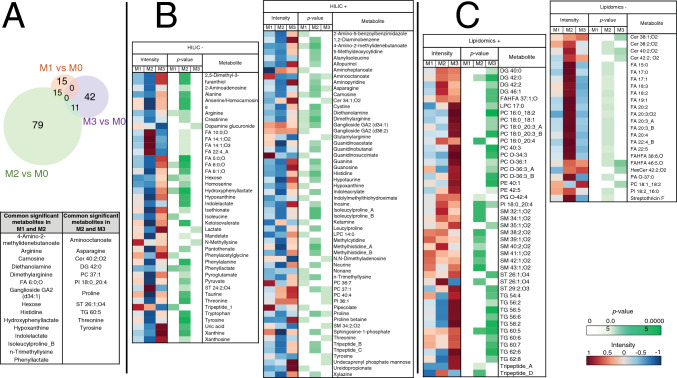
Plasma metabolite changes associated with PH and RV pressure overload. Plasma samples from pig models M1 (chronic postcapillary PH by pulmonary vein banding), M2 (chronic PH by aorto-pulmonary shunting), M3 (RV pressure overload by pulmonary artery banding), and M0 (sham procedure) were analyzed by LC–MS-based metabolomics (lipidomics and HILIC analysis in positive and negative ionization modes). Metabolites showing significant alterations in the three pathologic groups (M1, M2, and M3) versus M0 were annotated. **A** Venn-diagram showing the number of significantly altered metabolites in each pathologic group versus M0 (Mann–Whitey *U* test; *p* < 0.05). Metabolites commonly altered in more than one disease model are listed in the table. **B** Heatmap and *p*-value matrix of the significantly altered metabolites detected by HILIC in each disease model. *Intensity* columns indicate the abundance of each metabolite; *p-value* columns list the *p*-value for each metabolite (Mann–Whitey *U* test; *p* < 0.05). **C** Heatmap and *p*-value matrix of the significantly altered metabolites detected by lipidomics in each disease model. *Intensity* columns indicate the abundance of each metabolite; *p-value* columns show the *p*-value for each metabolite (Mann–Whitey *U* test; *p* < 0.05). Note: the figure includes only those annotated metabolites recognized by Metaboanalyst (REF). + , positive ionization mode; −, negative ionization mode; *Cer* ceramide; *DG* diglyceride; *FA* fatty acid; *FAHFA* fatty acid esters of hydroxy fatty acids; *HexCer* hexosylceramide; *HILIC* hydrophilic interaction liquid chromatography; *LPC* lysophosphatidylcholine; *PA* phosphatidic acid; *PC* phosphatidylcholine; *PE* phosphatidylethanolamines; *PG* phosphatidylgylcerol; *PI* phosphatidylinositol; *SM* sphingomyelin; *ST* sterol lipid; *TG* triglyceride

Metabolomics analysis revealed that, compared with M0, M1 showed significant alteration in 73 features from which 30 metabolites were annotated, M2 in 265 features from which 105 metabolites were annotated, and M3 in 117 features from which 53 metabolites were annotated (Fig. 1A). From them, 15 altered metabolites were common to both PH models (M1 and M2) (including l-arginine, histidine, and carnosine); 11 common to M3 and M2; and none between M3 and M1.

**Fig. 2:**
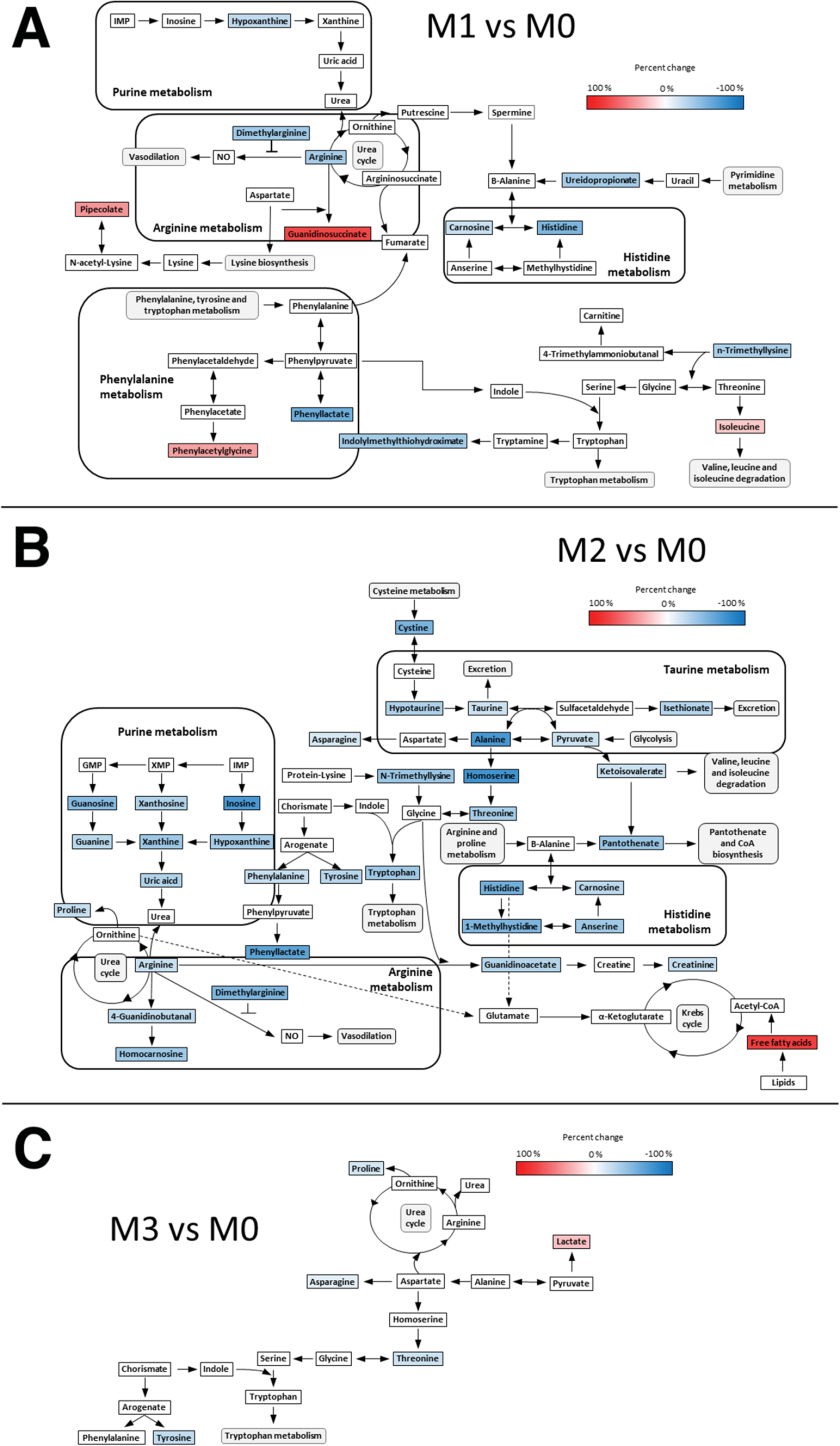
Selected altered metabolic pathways and their interrelations in the pathologic models. **A** M1 vs M0. **B** M2 vs M0. **C** M3 vs M0. Colored metabolites were detected by HILIC or lipidomics LC-QTOF-MS. The color of each metabolite reflects its percentage change vs M0. Note: the figure includes only those detected metabolites that could be linked to specific metabolic pathways

The 30 altered metabolites in M1 belonged to 15 subclasses (Supplementary Table 5) and included significant reductions in arginine and histidine (Figs. 1B, 2A). In contrast, glycosphingolipids (gangliosides) were increased (Fig. 1B). M2 showed the greatest metabolic alteration, with the 105 significantly altered compounds belonging to 38 subclasses (Supplementary Table 6). Metabolites of the arginine and histidine pathways were again significantly reduced, accompanied here by significant reductions in taurine and related compounds such as alanine, pyruvate, and isethionate, as well as purine degradation metabolites such as guanine, inosine, and xanthine (Figs. 1B, 2B). Conversely, there was an increase in circulating lipids, including free fatty acids (FA) and sphingomyelins (SM) (Fig. 1B, C). The analysis of free FA composition revealed higher levels of several omega-6 fatty acids, such as linoleic acid, gamma-linolenic acid, dihomo-gamma-linolenic acid, and arachidonic acid. The 53 metabolites significantly altered in M3 correspond to 16 subclasses (Supplementary Table 7), with most altered compounds being lipid metabolites such as triglycerides (TG), ceramides (Cer), and phosphatidylcholines (PCs), which tended to be increased. Animals in the M3 group showed no significant alterations in the arginine, histidine, taurine, or purine pathways (Fig. 2C).

### Proteomics analysis shows differential regulation of the complement and coagulation pathways in the three experimental models

**Fig. 3:**
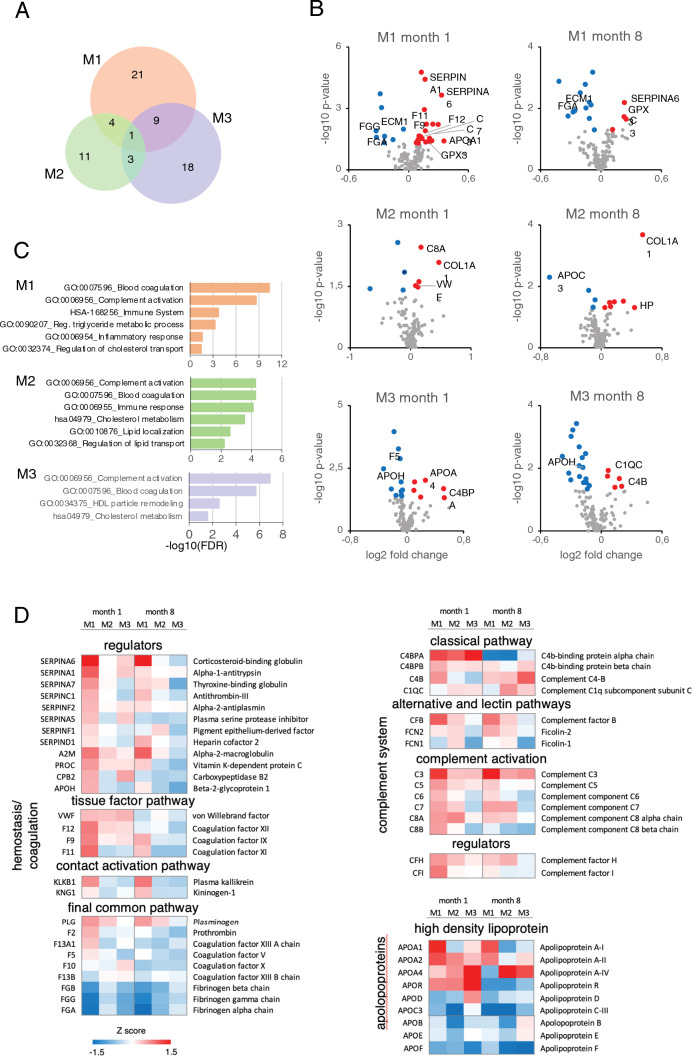
Quantitative proteomics detection of alterations to plasma proteins related to hemostasis/coagulation, the Complement system, and lipid binding (apolipoproteins) in pig models of RV pressure overload. Plasma samples of 16 randomly selected animals (4 per experimental model) were analyzed by multiplexed isobaric labeling and LC–MS/MS, and the quantitative data were analyzed with the WSPP model to detect protein abundance changes in the three disease models (M1, M2, and M3) with respect to the control group (M0). A total of five blood samples were processed per animal: one baseline sample extracted before surgery and four samples extracted at 1 and 8 months after surgery (Supplementary Table 1). **A** Venn-diagram showing the number of significant protein abundance changes found in the three disease models versus the control group (Student *t*-test; *p* < 0.05). **B** Volcano-plots highlighting the significant protein abundance changes found in the three disease groups versus the control group at 1 and 8 months after surgery. Red and blue circles indicate increased and decreased protein abundance, respectively. The corresponding gene names for the specifically altered proteins in each group are indicated. The complete set of proteins quantitated in this comparative analysis is provided in Supplementary Table 8. **C** Functional enrichment analysis of the proteins found altered in the three disease models (M1, orange; M2, green; and M3, purple) versus the control group (M0). An FDR was calculated based on the *p*-value obtained using the Benjamini–Hochberg procedure. The figure shows only significantly enriched GO terms (−log10(FDR) > 1.3, which corresponds to FDR < 0.05). The complete set of enriched GO terms is shown in Supplemental Table 9. **D** Relative protein quantification in the Hemostasis/coagulation, Complement system, and Apolipoprotein functional categories for the three disease models. The heatmaps represent the standardized protein variable (*Z*-score, see color scale at the bottom) calculated with log2 fold change values. *FDR* false discovery rate; *LC–MS/MS* liquid chromatography coupled to tandem mass spectrometry; *PH* pulmonary hypertension; *WSPP* weighted spectrum, peptide and protein

Proteomics analysis of plasma samples revealed altered protein profiles at 1 and 8 months after surgery in all three experimental groups, with significant changes with respect to M0 in 35, 19, and 31 proteins in M1, M2, and M3, respectively (Fig. 3A and Supplementary Table 8). Significant protein abundance alterations specific to M1 included SERPINA1; SERPINA6; coagulation factors IX (F9), XI (F11), and XII (F12); complement components C3 and C7; apolipoprotein A-1 (APOA1); extracellular matrix protein 1 (ECM1); and glutathione peroxidase 3 (GPX3). M2 showed specific alterations in haptoglobin (HP); collagen alpha-1(I) chain (COL1A1); complement component C8A, von Willebrand factor (VWF); and apolipoprotein C-III (APOC3). M3 showed specific alterations in coagulation factor V (F5); beta-2-glycoprotein 1 (APOH); the complement components C4B, C4BPA, and C1QC; and apolipoprotein A-IV (APOA4) (Fig. 3B). A functional enrichment analysis revealed significant overrepresentation of biologic processes related to blood coagulation, complement activation, and lipid metabolism (Fig. 3C and Supplementary Table 9).

To gain further insight into the functional alterations produced by PH, we analyzed the abundance changes of all proteins in these categories, using a curated protein classification based on a previous study [14]. Proteins involved in *Hemostasis/coagulation* were predominantly increased in M1 (Fig. 3D, Left). They included several serpin regulators and other regulator proteins such as alpha-2-macroglobulin (A2M), vitamin K-dependent protein C (PROC), carboxypeptidase B2 (CPB2), and beta-2-glycoprotein 1 (APOH), as well as coagulation factors implicated in the tissue factor pathway (extrinsic pathway), and prothrombin (F2) and coagulation factor XIIIa (F13A1), involved in the final common pathway. Also increased were 2 proteins of the contact activation pathway (intrinsic pathway), plasma kallikrein (KLKB1) and kininogen-1 (KNG1).

M1 and M2 showed a coordinated increase in the relative abundance of many *Complement system* proteins, mainly those involved in complement activation (C3, C5, C7, C6, C8A, and C8B) (Fig. 3D, Right). M1 and M2 also showed increases in complement factor H (CFH), a prominent modulator of complement activation, and complement factor B (CFB), a component of the alternative pathway.

The protein abundance analysis also revealed widespread (but uncoordinated) changes in the abundance of *Apolipoproteins* in the three models (Fig. 3D, Right). Notable changes included an increase in M1 of apolipoprotein A-I (APOA1), the main component of high-density lipoproteins, and a general reduction of low-density lipoproteins in all three models.

Multiplex immunoassay analysis confirmed significant abundance changes in coagulation and complement system components, particularly in M1 (Supplementary Fig. 2). CILP1 and NT-proBNP showed a moderate correlation (*R* = 0.52, *p* = 0.041) and significantly differed among groups (*p* = 0.021 and 0.026). In the post-hoc analysis, CILP1 was significantly higher in M1 whereas NT-proBNP was increased in M2, compared with M0 (Supplementary Fig. 3).

### RV failure is inversely associated with arginine, histidine, taurine and purine metabolism and positively related to complement system activation and circulating FA

**Fig. 4:**
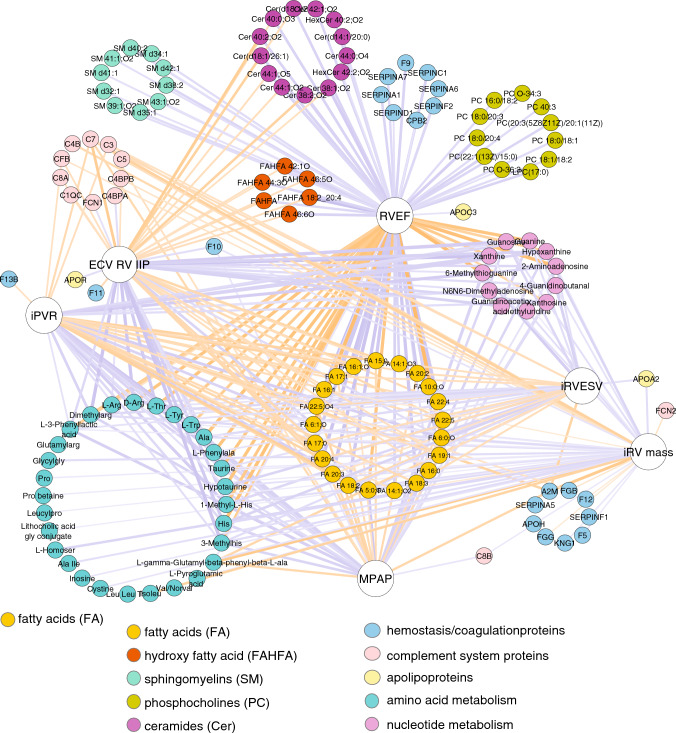
The maladaptive RV hypertrophy network. Significant (*p* < 0.05) relationships revealed by Pearson correlation analysis between quantitative data from omics (proteins, metabolites, and lipids), hemodynamics (indexed PVR and mean PAP), CMR imaging (indexed RV mass, indexed RV end-systolic volume, RV ejection fraction, and ECV at the inferior RV insertion point). Nodes representing protein, metabolite, and lipid components are color-coded based on functional attributes or chemical composition. Positively and negatively correlating nodes are denoted using orange and purple connecting lines, respectively, where line thickness is proportional to the absolute value of the Pearson correlation coefficient

**Fig. 5:**
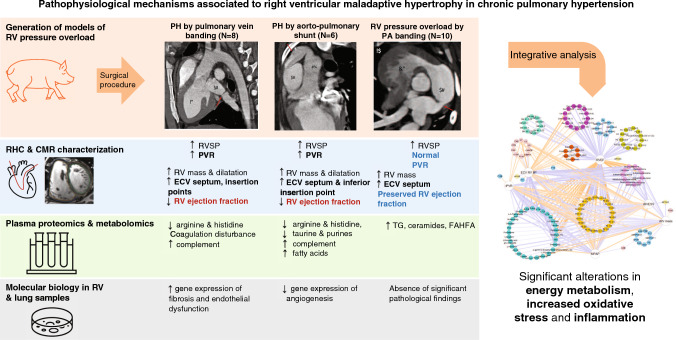
The pathophysiological mechanisms associated with right ventricular maladaptive hypertrophy in PH were investigated in three large-animal surgical models: postcapillary PH (M1), PH secondary to aorto-pulmonary shunting (M2), and RV pressure overload by PA banding (thus without PH). These models were compared with sham-operated animals (M0). The columns summarize the main outcomes obtained for each disease model studied by RHC hemodynamics and CMR (RV adaptation), RV and lung molecular biology, and unbiased plasma proteomics and metabolomics. These findings formed the basis of an integrative analysis combining omics with RV performance and hemodynamic data on PH severity. *ECV* extracellular volume; *IIP* inferior insertion point; *PA* pulmonary artery; *PH* pulmonary hypertension; *PVR* pulmonary vascular resistance; *RHC* right heart catheterization; *RV* right ventricle; *RVSP* right ventricular systolic pressure; *TG* triglycerides

An integrative analysis revealed that, in the scenario of PH, the hemodynamic severity (assessed by PAP and PVR) was negatively associated with arginine, histidine, taurine, and purine metabolism and positively associated with complement system activation and circulating FAs; and RV maladaptive hypertrophy (assessed by RV end-systolic volume, RV ejection fraction, and ECV at the inferior RV insertion point) was inversely associated with plasma arginine, taurine, histidine, and purines and positively correlated with plasma coagulation factors, complement system components, and circulating lipids (FA, FAHFA, SM, PC, ceramides, and cholesterol derivatives) (Supplemental Table 10, Figs. 4). The association between the main identified metabolites and RV ejection fraction, as the most relevant parameter of RV performance, remained after adjusting by weight (Supplementary Table 11). NT-proBNP levels showed a modest correlation with RV ejection fraction (*R* = −0.409) but CILP1 did not (*R* = −0.319, *p* = 0.229). Figure 5 summarizes the main outcomes obtained for each disease model studied by RHC hemodynamics and CMR (RV adaptation), RV and lung molecular biology, and unbiased plasma proteomics and metabolomics.

### Plasma omics findings match gene expression changes in myocardium and lung samples

Gene expression analysis of RV myocardium samples revealed patterns suggestive of more severe myocardial damage in M1 and M2 than in M3 (higher involvement of the arginine-NO pathway, increased fibrosis, endothelial dysfunction, and reduced angiogenesis) (Supplementary Fig. 2). As evidence, both PH models showed significantly reduced gene expression of arginine N-methyltransferases compared with M0 (PRMT1 and PRMT2 in M1; PRMT1 in M2). M1 samples also showed significant reductions in tissue inhibitor of metalloproteinases (TIMP-2) and stromal cell-derived factor 1 (SDF-1), whereas M2 samples showed significant reductions in sphingosine-1-phosphate receptor (S1PR3), VEGF-2, APOE, and intercellular adhesion molecule 1 (ICAM1). In contrast, M3 samples showed no major differences in gene expression relative to M0 (TIMP-4 was slightly reduced and complement component 5a receptor 1 [C5AR1] slightly increased).

Reflecting the absence of PH in M3, the lung parenchyma of animals in this group showed no gene expression changes. In contrast, M1 samples showed overexpression of the pro-oxidant enzyme NOX5 and downregulation of the anti-fibrotic MMP2, APOE, and the kallikrein cascade components KLKB1 and KNG1. Similarly, M2 samples showed overexpression of the pro-fibrotic MMP9 and downregulation of TIMP4 and the peroxide-depleting enzyme CAT in lung parenchyma (Supplementary Fig. 4).

## Discussion

RV adaptation to pressure overload remains the main prognostic factor in patients with chronic PH [10], albeit poorly understood. Our analysis, including imaging, proteomics, and metabolomics studies in pigs (Fig. 5), produced a number of key findings. (1) RV adverse remodeling and dysfunction (assessed by CMR) was greater in the presence of PH (M1 and M2) than in animals with isolated RV pressure overload (M3), even though pigs in the M3 group had larger increases in RV systolic pressure and more pronounced RV hypertrophy. (2) Unlike the isolated pressure overload model, the PH models were associated with deficiency in metabolites such as arginine, histidine, taurine, and purines, together with altered abundance of proteins related to the coagulation pathways and complement system activation. (3) All three models of RV pressure overload showed systemic increases in lipid compounds although of different category. (4) In the integrative analysis, the parameters most strongly associated with maladaptive RV hypertrophy in PH were deficiencies in arginine, histidine, taurine, and purine metabolic pathways, complement system activation, and increased circulating FAs.

Although previous experimental studies have investigated potential mechanisms of RV failure in PH [1], these were performed in small animal models, and thus hemodynamic evaluation of PH severity was limited to RV systolic pressure; moreover CMR analysis was very seldom included. Other drawbacks of small animal studies are the induction of PH by infusion of toxins such as SU5416 which could modify signaling pathways in the RV, and the acquisition of results after a short follow-up. Finally, previous studies have generally focused on the RV myocardium, and have not investigated potential mediators leading to myocardial injury, which may have an origin in the pulmonary vasculature.

Our metabolomic analysis showed that PH was associated with significant changes in energy metabolism and increased oxidative stress. In line with prior findings in PAH patient samples [16, 30, 32], we found decreased plasma arginine in both PH models, correlating with maladaptive RV hypertrophy in vivo. Decreased plasma arginine is an indicator of increased arginase activity, which leads to competition with nitric oxide (NO) synthase and hence reduced NO [25]. Indeed, previous studies have shown an inverse association between plasma arginine and PAP [19], and also prognosis [16, 32]. The two PH models also showed reduced myocardial expression of arginine *N*-methyltransferases, and M1 animals additional upregulation in lung parenchyma NOX5. These findings are in line with patient data showing PRMT1 downregulation and NOX5 overexpression in heart failure [22]. Moreover, PRMT1 ablation in mouse cardiomyocytes causes myocardial dysfunction [22].

The PH models also had altered histidine metabolism, indexed by significantly decreased plasma levels of anserine, 1-methyl-histidine, carnosine, and histidine. Decreased plasma histidine is a prognostic indicator in patients with heart failure [4], likely due to the shift from FA to glucose utilization in response to the histidine deficiency.

Animals with PH induced by aorto-pulmonary shunting (M2) showed a particularly pronounced effect on taurine metabolism, evidenced by reduced plasma levels of hypotaurine, taurine, alanine, and pyruvic acid. Taurine has an antioxidant effect mainly attributed to its mitochondrial protective effect, and low plasma taurine has been reported in patients with diabetic cardiomyopathy [6] and linked to cardiac remodeling in experimental models of taurine deficiency [18] Nevertheless, the most altered pathway in the aorto-pulmonary shunting PH model was the purine pathway, with marked decreases in 8 metabolites, including inosine, hypoxanthine, xanthine, guanine, and uric acid. These metabolites have been inversely linked to PAH and hemodynamic severity [13, 30] and have been proposed as prognostic indicators in PAH [17]. The more pronounced changes to taurine and purine metabolism in M2 than in M1 likely reflects a greater involvement of the pulmonary vasculature. The integrative analysis revealed that the taurine and purine pathway deficiencies were related to PH severity and RV maladaptive hypertrophy, suggesting that alterations to these pathways might be important in maladaptive RV remodeling associated with PH.

Lipidomics has seldom been used to investigate PH, and our study provides interesting data in this area. Plasma lipid concentrations were significantly increased in all three RV pressure overload models, thus suggesting an association with RV hypertrophy independently of PAP. However, the type of lipid affected differed between the models, with PCs and TGs increased in M3, FAs and SMs in M2, and only a few glycosphingolipids in M1. Interestingly, the only lipids showing a significant correlation with PH severity and maladaptive RV hypertrophy were free FAs. In PH, FA accumulation has been linked to the disruption of β-oxidation in mitochondria and peroxisomes [5] and the resulting metabolic switch from lipid oxidation to glycolysis and lactate-producing anerobic metabolism, causing a gradual export of lipids into the bloodstream and increasing circulating free FA concentrations [3]. Impaired myocardial β-oxidation and increased glucose utilization result in less efficient ATP production, decreased contractile performance, and progression toward RV failure [31]. Chronic RV failure is frequently associated with cachexia, particularly in patients with concomitant LV heart failure, which aggravates prognosis. The large differences in weight gain between animals with PH (M1 and M2) compared to animals with isolated pressure overload (M3), together with the differences in metabolism, open new opportunities to understand the genesis and differences in the establishment of cachexia among patients.

The proteomics analysis revealed a significant overrepresentation of biologic processes related to blood coagulation, complement activation, and lipid metabolism, suggesting these as functional proteomic footprints of PH-derived RV failure in plasma. Whereas changes in complement activation were observed in both PH models, only the postcapillary PH model showed evidence of an enhanced procoagulant state, which might reflect greater venous stagnation in postcapillary PH. Complement activation correlated significantly with PH hemodynamic severity and maladaptive RV hypertrophy in the integrated interaction network, thus suggesting that inflammation plays an important role in RV dysfunction secondary to PH. This inflammation and oxidative stress in PH may have different sources. Systemic increase in free FAs, observed in M2, especially arachidonic acid, are precursors of pro-inflammatory and pro-oxidant eicosanoids, such as prostaglandins, leukotrienes, and thromboxanes [11]. On the other hand, the decreased arginine and impaired NOS activity may reduce NO production and increase superoxide generation [29]. Remarkably, the model of pure RV pressure overload, despite developing a much more pronounced RV hypertrophy, maintained normal RV function and showed little of the alteration of pathologic biologic processes seen in the presence of PH. Instead, M3 animals presented an adaptive RV hypertrophy with markers that have been reported as cardioprotective, such as increased expression of C5aR1 [28]. CILP1 and NT-proBNP, peptides previously linked to maladaptive RV remodeling [12] were significantly increased in M1 and M2, respectively, which confirmed the higher specificity of CILP1 over NT-proBNP for RV versus LV involvement [12, 27]. However, we only found a modest correlation between NT-proBNP levels and RV ejection fraction but not for CILP1. This is possibly because all animals had a RV ejection fraction higher than 42% and CILP1 is a marker of more advanced RV remodeling.

Our study is limited by the sample size, which might have rendered the analysis underpowered to assess differences in some parameters. Moreover, proteomics analyses were performed on a smaller cohort than metabolomics. The reason was that proteomics methodology (based on isobaric labeling), can minimize technical variability and allows very precise quantification, while metabolomics is undertaken in label-free mode, with independent runs for each sample. In addition, since all proteomics and metabolomics analyses were performed on plasma samples, it is difficult to determine if they reflect metabolomic changes originating in the lung or RV. However, this limitation is to some degree overcome by the use of the pure RV pressure-overload model, without PH, and the targeted molecular biologic analysis of RV and lung parenchyma samples. Finally, our study was performed in male castrated animals which may limit generalization of these results.

In conclusion, our study integrating imaging and omics in large-animal experimental models demonstrates that, beyond pressure overload, metabolic alterations play a relevant role in RV dysfunction in PH. These findings open up new research avenues for the identification of novel therapeutic targets.

### Supplementary Information

Below is the link to the electronic supplementary material.

Supplementary file1 (DOCX 2309 KB)

## Data Availability

Please see Online Appendix for proteomic and metabolomic data availability at dedicated repositories.

## Abbreviations

CILP-1: Cartilage intermediate layer protein
CMR: Cardiac magnetic resonance
CT: Computed tomography
ECV: Extracellular volume
FA: Fatty acids
FAHFA: Fatty acid esters of hydroxy fatty acids
FDR: False discovery rate
HILIC: Hydrophilic interaction liquid chromatography
LVEDP: Left ventricular end-diastolic pressure
MOLLI: Modified Look-Locker inversion-recovery
NT-proBNP: N-terminal pro-brain natriuretic peptide
PA: Pulmonary artery
PAH: Pulmonary arterial hypertension
PAP: Pulmonary artery pressure
PAWP: Pulmonary artery wedge pressure
PC: Phosphatidylcholine
PH: Pulmonary hypertension
PVR: Pulmonary vascular resistance
QTOF: Quadrupole time-of-flight
ROI: Region of interest
RV: Right ventricle
SM: Sphingomyelin
WU: Wood units
