# Supplementary material for: Metabolic changes contribute to maladaptive right ventricular hypertrophy in pulmonary hypertension beyond pressure overload: an integrative imaging and omics investigation
Source: Basic Res Cardiol. 2024 Mar 27;119(3):419–33. doi: 10.1007/s00395-024-01041-5 (PMC11143050; doi:10.1007/s00395-024-01041-5)
Supplement: Supplementary file 1 — Supplementary file1 (DOCX 2309 KB) [file 395_2024_1041_MOESM1_ESM.docx]

**ONLINE APPENDIX**

**Metabolic changes contribute to maladaptive right ventricular hypertrophy in pulmonary hypertension beyond pressure overload**

***An integrative imaging and omics investigation***

Inés García-Lunar et al.

1. SUPPLEMENTARY METHODS

1.1 Periprocedural anesthesia and analgesia

1.2 Right heart catheterization

1.3 Advanced imaging studies

1.3.1 Cardiac magnetic resonance studies

1.3.2. Computed tomography studies

1.4. Metabolomics

1.5. Proteomics

1.6. Data availability

1.7. Molecular biology analysis

1.8. Sample size estimation

3. SUPPLEMENTARY TABLES

4. SUPPLEMENTARY FIGURES

5. SUPPLEMENTARY REFERENCES

**1. SUPPLEMENTARY METHODS**

**1.1. Periprocedural anesthesia and analgesia**

Before any procedure, anesthesia was induced by intramuscular injection of ketamine (20 mg/kg), xylazine (2 mg/kg), and midazolam (0.5 mg/kg), together with buprenorphine (0.3 mg/kg) for analgesia, and the animals were intubated. All CMR and hemodynamic measures were obtained during spontaneous ventilation, anesthesia maintained with intravenous midazolam (0.2 mg/kg/h), and continuous electrocardiographic and oximetric monitoring.

**1.2. Right heart catheterization**

Right heart catheterization (RHC) was performed with a Swan-Ganz catheter inserted via the right femoral vein following a standardized methodology that has been previously described(1, 2, 3, 4, 5). Hemodynamic measures included mean PAP, pulmonary artery wedge pressure (PAWP) at end-expiration, and cardiac output assessed by thermodilution. Left ventricular end-diastolic pressure (LVEDP), measured with a pigtail catheter, was used as a surrogate of left atrial pressure. PVR was calculated as the difference between mean PAP and LVEDP divided by the cardiac output in Wood units (WU). Cardiac output was indexed by body surface area estimated with the Brody formula, and the PVR index was calculated (RVP*m^2^).

**1.3. Advanced imaging studies**

CMR studies were performed immediately after RHC. CMR assessment included cine, flow imaging, delayed enhancement, and T1-mapping sequences.

**1.3.1. Cardiac magnetic resonance studies**

All CMR studies were performed with a 3.0 T magnet (Achieva-Tx, Philips Medical Systems, The Netherlands) equipped with a 32-channel cardiac phased-array surface coil and retrospective electrocardiographic gating during spontaneous ventilation. The detailed CMR acquisition protocol has been reported in detail elsewhere(2, 3, 4, 6). Briefly, ventricular volumes and function were assessed from steady-state free precession cine sequences acquired in 10–15 contiguous short axis slices covering both ventricles from base to apex with 30 cardiac phases each. Two-dimensional flow imaging (phase contrast) was performed perpendicular to the main pulmonary artery (PA) with a velocity-encoded gradient echo sequence using the minimum upper velocity limit without signal aliasing.(1) Gadolinium contrast (gadopentetate dimeglumine, Bayer Healthcare Pharmaceuticals, Whippany, New Jersey) was administered at a dose of 0.1 mmol/kg for late gadolinium enhancement sequence and 0.0011 mmol/kg/min for a 20-min infusion started after a 15-min delay for equilibrium contrast imaging, with a cumulative contrast dose of 0.122 mmol/kg(3). The T1 mapping sequence (modified Look-Locker inversion-recovery [MOLLI]) was acquired immediately before contrast administration and after equilibrium in a short-axis view at the level of the papillary muscles.

CMR acquisitions were analyzed with specialized software (ISP Portal, Philips Healthcare, The Netherlands). The methodology for the analysis has been described previously in detail(1, 2, 3, 4, 5, 6, 7). On cine images, biventricular endocardial contours were manually traced at end-diastole and end-systole, and Simpson’s method was used to calculate volumes and ejection fraction. RV trabeculations were included within the blood pool, and the interventricular septum was assigned to the LV mass. Similarly, the inner contour of the main PA was outlined in each cardiac phase to quantify the minimum and maximum areas, mean PA velocity, and RV stroke volume. Ventricular volumes and masses and PA areas were similarly adjusted to body surface area. PA compliance was calculated as the ratio of stroke volume and PA pulse pressure. RV-arterial coupling—the ratio of PA effective elastance (Ea) to RV maximal end-systolic elastance (Emax) (Ea/Emax)—was estimated as ([mean PAP - LVEDP]/RV stroke volume index/[mean PAP/RV end-systolic volume index]). Regions of interest (ROIs) were drawn on T1 maps at the myocardial anterior and inferior RV insertion points, the interventricular septum and LV lateral wall, and the LV cavity blood pool before contrast administration and after equilibrium. When drawing ROIs, special care was taken to avoid the tissue interface between myocardium and blood and thus partial volume averaging by blood contamination. T1 mapping images were acquired with high in-plane resolution to facilitate drawing the ROI inside the myocardium, and heart rate was updated before every MOLLI acquisition to define the correct trigger delay to minimize spatial misregistration. Native T1 values were obtained from the pre-contrast T1 map, and extracellular volume (ECV) values were calculated as previously described.(3)

**1.3.2. Cardiac computed tomography**

CT studies using a 64-slice scanner (Brilliance Philips) with retrospective electrocardiographic gating were performed to confirm the correct surgical execution of the experimental models, i.e., stenosis of the banded pulmonary veins in M1, shunt permeability in M2, and PA stenosis in M3. Nonionic iodinated contrast agent (30–40 mL; Isovue 300) was injected through a peripheral vein at a rate of 4 mL/s with a power injector followed by a 30 mL saline flush. A bolus tracking technique was used to appropriately trigger image acquisition once attenuation in the left atrium (M1) or RV (M2 and M3) reached a preset threshold of 100 HU.

CT images were postprocessed using dedicated software (ISP Portal, Philips Healthcare, The Netherlands), and postprocessing included multiplanar, volume-rendered, and shaded-surface display reconstructions. Shunt maximal orthogonal diameters and cross-sectional area were measured at end-diastole. PA percent stenosis was calculated using the North American Symptomatic Carotid Endarterectomy Trial (NASCET) equation from cross-sectional pre-banding and maximal stenosis areas.(8)

**1.4. Metabolomics:**

For lipidomics and HILIC analyses, samples were acquired in positive and negative ionization modes.

**1.4.1. Lipidomics**

Lipidomics analyses were performed using a comprehensive method capable of detecting more than 42 different lipid classes, based on a gradient chromatography approach with mobile phases containing ammonium acetate and ammonium fluoride to enhance ionization (9). Data were recorded in scan mode, and tandem MS/MS analyses were performed to assist in lipid identification. Sample preparation consisted of deproteinization and lipid extraction from 40 μL of thawed plasma with 800 μL of a solvent mixture composed of methanol/methyl tert-butyl ether/chloroform (4:3:3, v/v) and the internal standards C17-sphinganine (0.8 ppm) and D-palmitic acid (3.75 ppm). The mixture was vortex-mixed for 30 s, shaken at 1000 rpm for 20 min at room temperature, and centrifuged for 5 min at 3000 rpm. The supernatant was then collected and transferred into an LC vial for analysis(10). Adequate quality control (QC) samples resulting from pooling all the plasma samples were prepared, as well as solvent blanks and sample preparation blanks.

Samples were analyzed in an Agilent 1290 Infinity II UHPLC system coupled to an Agilent 6545 quadrupole time-of-flight (QTOF) mass spectrometer in positive and negative ionization modes. Chromatography was performed with an Agilent InfinityLab Poroshell 120 EC–C18 column (3.0 × 100 mm, 2.7 μm) (Agilent Technologies). The mobile phases used for both positive and negative ionization modes consisted of (A) 10 mM ammonium acetate and 0.2 mM ammonium fluoride in 9:1 water/methanol, and (B) 10 mM ammonium acetate and 0.2 mM ammonium fluoride in 2:3:5 acetonitrile/methanol/isopropanol. The flow rate was held constant at 0.6 mL/min, and the column was maintained at 50°C throughout. The same chromatographic gradient was used for positive and negative ionization modes, which started at 70% B maintained for 1 min. B was then increased to 86% at minute 3.5 and maintained until minute 10. B was then increased to 100% B at minute 11 and maintained until minute 17. The starting conditions were recovered by minute 17, followed by a 2 minute re-equilibration(11). The injection volume was 2 µL for the analysis in negative ionization mode and 1 µL for the analysis in positive ionization mode.

Ion-source parameters in the mass spectrometer were as follows: gas temperature 200°C, flow rate 10 L/min, nebulizer pressure 50 psig, sheath gas temperature 300°C, and sheath gas flow rate 12 L/min. The capillary voltage was set at 3000 V for negative mode and 3500 V for positive mode, the fragmentor was set at 150 V, the skimmer voltage at 65 V, and the octopole radio frequency voltage at 750 V. Iterative-MS/MS fragmentation analyses were performed in the QC samples at the end of the run to assist with lipid identification. In total, 5 runs were done with the collision voltage set at 20 eV and 5 consecutive runs with the collision voltage set at 40 eV.

**1.4.2. HILIC**

For HILIC analysis, mobile phases for the positive ionization mode were based on ammonium formate at acidic pH, whereas for analysis in negative ionization mode, the mobile phases were ammonium acetate-based at basic pH(12). HILIC data were also recorded in scan mode and using tandem MS/MS fragmentation for compound identification. Sample preparation consisted of deproteinization of 100 µL of plasma with 300 µL of cold methanol, followed by vortex-mixing for 1 minute and centrifugation at 13200 rpm for 10 min. The supernatant was collected and transferred to an LC vial for analysis(12). Adequate QC samples resulting from pooling all the plasma samples were prepared, as well as solvent blanks and sample preparation blanks.

Samples were analyzed in an Agilent 1290 Infinity II UHPLC system coupled to an Agilent 6545 quadrupole time-of-flight (QTOF) mass spectrometer in positive ionization mode and in an Agilent 1290 Infinity II UHPLC system coupled to an Agilent 6560 ion mobility quadrupole time-of-flight (IM-QTOF) mass spectrometer in negative ionization mode. Chromatographic separation was performed in a Water XBridge® BEH Amide XP 2.5 µm, 2.1 mm x 100 mm Column XP (Waters, Milford, MA, USA).

For negative mode, the column was maintained at 50°C, and the mobile phases were made from a 100 mM ammonium acetate (pH = 9) stock solution. The mobile phase A was composed of 10 mM ammonium acetate and 2.5 µM InfinityLab deactivator additive (Agilent, P-N. 5191-4506) in water. Mobile phase B was composed of 10 mM ammonium acetate adjusted to pH = 9 in H_2_O/ACN (15:85, v/v) with 2.5 µM of the same deactivator. The flow rate was maintained at 0.250 mL/min throughout the analysis. The chromatographic gradient was 96% B at 0–2.0 min, 88% at 5.5–8.5 min, 86% at 9.0–14.0 min, 82% at 15—17.0, and 65% at 23.0 -24.0 min. At 24.5 min, the percentage of B was restored to the initial conditions and maintained until 29.0 min for column re-equilibration. Before each injection, the injection needle and the injection port were flushed with the proportions of mobile phases A and B matching the initial chromatographic conditions. The injection volume was set to 2 µL. The ion source was set with the following parameters: gas temperature 225°C, nebulizer gas flow rate 13 L/min, nebulizer gas pressure 35 psig, sheath gas temperature 350°C, sheath gas flow rate 12 L/min, capillary voltage 3500 V, fragmentor voltage 400 V, skimmer 45 V, and octopole radio frequency 750 V.

For positive mode, the column was kept at 25°C, and the mobile phases were prepared from a 100mM ammonium formate stock solution. Mobile phase A was composed of 10mM ammonium formate with 2.5 µM InfinityLab deactivator additive (Agilent, P-N. 5191-4506) and 0.1% formic acid. Mobile phase B was composed of 10 mM ammonium formate in H_2_O/ACN (10:90, v/v, using weighed acetonitrile) with 2.5 µM of the same deactivator. The flow rate was constant at 0.250 mL/min. The chromatographic gradient started at 98% B, maintained for 3 min. B was decreased to 70% at min 11, and to 60% at min 12. At minute 16, B was decreased again to 5% and maintained until min 18. At 19 min, the percentage of B was restored to the initial conditions and maintained until min 20 for column equilibration. The injection needle and the injection port were flushed with mobile phase B before each injection. The injected sample volume was 1 µL. The ion source was set with the following parameters: gas temperature225°C, nebulizer gas flow 6 L/min, nebulizer gas pressure 40 psig, sheath gas temperature 225°C, sheath gas flow 10 L/min, capillary voltage 3000 V, fragmentor voltage 125 V, skimmer 65 V, and octopole radio frequency 450V.

**1.4.3. Data analysis**

After data acquisition, the data files were inspected using Agilent MassHunter Qualitative B.10.0.2, and MassHunter Profinder B.10.0.2 was used for data alignment, deconvolution, and peak integration. After peak integration, statistical analysis was performed (see *Statistical analysis*), and significantly different features were annotated. Annotation was carried out by searching the m/z of significant features on the online tool CEU Mass Mediator,(13) and manual MS/MS spectral inspection was performed for further confirmation. Lipid Annotator software (11) (Agilent Technologies Inc., Santa Clara, CA, USA) was used to assist in lipid identification, and an internal HILIC library was used for the annotation of polar metabolites(12).

**1.4.4. Feature detection and annotation results.**

In positive ionization mode for lipidomics, 535 features were initially detected post-integration, which were reduced to 386 features after matrix cleaning, (CV in QC samples>20% removal, blank subtraction, duplicate features elimination). Afterwards, statistical significance was observed for 87 of these features within at least one of the three groups, and 44 features were successfully annotated. In the context of negative ionization mode for lipidomics, an initial detection of 390 features was made following integration, resulting in 248 features after matrix cleaning. Among these, statistical significance was found for 61 features within one of the three groups, and 24 features were annotated.

In HILIC for positive ionization mode, 939 features were initially detected post-integration, ultimately narrowing down to 787 features after matrix cleaning. In this case, statistical significance was observed for 78 features within at least one of the three groups, and 58 features were annotated. Lastly, HILIC in negative ionization mode retained 923 features following integration, with 484 compounds remaining after matrix cleaning. Out of these, statistical significance was found for 75 features within one of the three groups, and 40 features were annotated.

**1.5. Proteomics**:

Proteomics analysis was performed in plasma samples obtained at baseline and at 1- and 8- months post intervention from a randomly selected subset of 16 pigs: 4 animals subjected to pulmonary vein banding (M1), 4 animals subjected to aorto-pulmonary shunting (M2), 4 animals subjected to artery banding (M3), and 4 animals subjected to the sham intervention (M0).

**1.5.1. Digestion of plasma samples**. Plasma samples were subjected to on-filter tryptic digestion using FASP technology (Expedeon). Briefly, the samples (100 μg) were diluted in urea solution (US, 8 M urea in 100 mM Tris-HCl at pH 8.5) and loaded onto the filters. After centrifugation and a washing step with US, oxidized Cys residues were reduced with 50 mM dithiothreitol (GE Healthcare) in US for 1 h at room temperature. The samples were then centrifuged, washed with US, and subsequently alkylated with 50 mM iodoacetamide (Sigma) in US for 1 h at room temperature in the dark. Thereafter the filters were washed 3 times in US and 3 times in ABC buffer (50 mM ammonium bicarbonate, pH 8.8). Proteins were digested with sequencing grade trypsin (Promega) in a 1:40 (w/w) trypsin:protein ratio at 37°C overnight with gentle agitation. The resulting tryptic peptides were recovered by addition of 40 μl ABC and centrifugation at 10,000 rpm for 5 min, followed by addition of 50 μl 500 mM NaCl and centrifugation at 13,000 rpm for 15 min. Trifluoroacetic acid was added to a final concentration of 1%, and the peptides were desalted on C18 Oasis HLB extraction cartridges (Waters Corporation, Milford, MA, USA) and dried-down.

**1.5.2. Isobaric peptide labeling**

The eluted, cleaned-up peptides were subjected to stable isotope labeling using tandem mass tags (TMT; Thermo Fisher Scientific, Bremen, Germany) according to the manufacturer’s protocol. A total of 8 TMT experiments were performed. (**Supplementary Table 1**). The differentially tagged peptide samples were then appropriately pooled and desalted on Waters Oasis HLB C18 cartridges.

**1.5.3. LC-MS/MS analysis**

The labeled peptide samples were analyzed in an Ultimate 3000 HPLC system (Thermo Fisher Scientific) coupled via a nanoelectrospray ion source (Thermo Fisher Scientific) to a Q Exactive HF mass spectrometer (Thermo Fisher Scientific). C18-based reverse phase separation was performed using a PepMap 100 5 μm 0.3 × 5 mm C18 column as the trapping column (Thermo Fisher Scientific) and a PepMap RSLC C18 EASY-Spray 50 cm × 75 μm ID column as the analytical column (Thermo Fisher Scientific). Peptides were loaded in buffer A (0.1% (v/v) formic acid in water) and eluted with a linear gradient consisting of 0-21% buffer B (100% acetonitrile, 0.1% (v/v) formic acid) for 300 min and 21–90% B for 5 min at a flow rate of 200 nl/min. Mass spectra were acquired in a data-dependent manner. MS spectra were acquired in the Orbitrap analyzer using full ion-scan mode with a 400-1500 m/z range and 70,000 FT resolution. The automatic gain control target was set at 2 x 10^5^ with 50 ms maximum injection time. MS/MS was performed using the top-speed acquisition mode with a 3 s cycle time. HCD fragmentation was performed at 30% of normalized collision energy, and MS/MS spectra were analyzed at a 60,000 resolution in the Orbitrap.

**1.5.4. Protein identification**

Proteins were identified using the SEQUEST HT algorithm integrated in Proteome Discoverer 2.1 (Thermo Fisher Scientific). MS/MS scans were matched against a concatenated protein database containing human and pig sequences (March 2019 release) and the corresponding inverted sequences. Database searching parameters were as follows: trypsin digestion with a maximum of 2 missed cleavage sites; precursor mass tolerance 800 ppm; fragment mass tolerance 0.02 Da. Fixed modifications were TMT modification at the N-terminus and Lys and Cys carbamidomethylation. Met oxidation was set as a variable modification. The false discovery rate (FDR) for peptide identification was calculated using the probability ratio method after a 15 ppm precursor mass tolerance postfiltering(14, 15).

**1.5.5. Protein quantification and statistical analysis**

Quantitative information was extracted from the intensity of TMT reporter ions. Protein abundance changes were assessed with iSanXot (16), a software application developed in our laboratory for the statistical analysis of high-throughput, quantitative proteomics experiments based on the WSPP statistical model.(17, 18) For every LC-MS/MS scan, iSanXot calculates a relative quantitative value, log2 (A/B), using the TMT reporter ion intensities from sample A (after surgery) and baseline sample B (before surgery) for each animal. Peptide log2 ratios were calculated as the weighted average of their corresponding scans; likewise, proteins were quantitated using the weighted average of their corresponding peptides. This model assigns to each protein a standardized variable (Z-score), defined as the mean-corrected log2-ratio expressed in standard deviation units. The results obtained for every biological replicate were then integrated into a weighted protein average as described (15,17) to determine statistically significant protein abundance changes between groups based on the Student *t* test (p < 0.05). Functional enrichment analysis was performed with String (<https://string-db.org/>),(19) and the FDR was calculated based on the p-value obtained using the Benjamini–Hochberg procedure for each Gene Ontology category.

**1.6. Data availability**

The raw LC-MS/MS files and tables with protein quantification data have been deposited with the ProteomeXchange Consortium via the PRIDE partner repository(20) with the dataset identifier PXD041643. Metabolomic data are available at the NIH Common Fund’s National Metabolomics Data Repository (NMDR) Metabolomics Workbench website, [https://www.metabolomicsworkbench.org](https://urldefense.com/v3/__https:/www.metabolomicsworkbench.org__;!!D9dNQwwGXtA!RetPPiziyrkaUVW4lIC0pGwKnwCyWOA6tXrb-21jGR9PZ7BtZywiQIvleFlu1e9dKwlfX4fBg1pjryiJFc4UIhhWAqYQsA$), with the ID ST002568. The data can be accessed directly via the Project DOI [http://dx.doi.org/10.21228/M8S726](https://urldefense.com/v3/__http:/dx.doi.org/10.21228/M8S726__;!!D9dNQwwGXtA!RetPPiziyrkaUVW4lIC0pGwKnwCyWOA6tXrb-21jGR9PZ7BtZywiQIvleFlu1e9dKwlfX4fBg1pjryiJFc4UIhiAPooh1g$).(21)

**1.7. Molecular biology analysis**

**1.7.1. Gene expression in heart and lung tissues**

The expression of mRNA encoding proteins linked to major pathways identified in the proteomics and metabolomics analyses was determined by quantitative real-time PCR (qPCR). Total RNA was isolated from RV myocardium and lung parenchyma using a column-based purification kit (PureLink™️, Thermo Fisher, Waltham, MA, USA). Equal amounts of total RNA (4 µg) from each preparation were reverse transcribed to complementary DNA (cDNA) with the High-Capacity cDNA Reverse Transcription Kit (Applied Biosystems, Thermo Fisher, Waltham, MA, USA). The cDNA of 78 genes was quantified in each sample in an ABI Viia7 Real-Time PCR System using the Sybr Green-based methodology (Applied Biosystems, Thermo Fisher, Waltham, MA, USA). Primers for each gene were based on the porcine mRNA sequence (**Supplementary Table 2**). Expression of mRNA for beta-glucuronidase and glyceraldehyde-3-phosphate dehydrogenase (GAPDH) were used as endogenous controls for RNA input and reverse transcription efficiency. The number of complementary DNA transcript copies in each tissue from M1, M2, and M3 samples was established relative to the reference sample (M0) and expressed as a fold change.

**1.7.2. Plasma protein concentration**

Plasma concentrations of proteins implicated in the coagulation and complement system cascades were determined with multiplexed bead-based immunoassays (R&D Systems, Inc. Minneapolis, MN, USA). The concentration of each biomarker was determined with a Luminex™200™ Instrument (Invitrogen, Thermo Fisher Scientific, Waltham, MA, USA), and data were reported as pg/mL.

**1.7.3. Additional analyses**

Plasmatic levels of cartilage intermediate layer protein (CILP)1 were determined using a high-sensitivity ELISA (human CILP1 ELISA kit, Cusabio Techology, Wuhan, China) with a detection range of 93.75–6000 pg·mL−1, a minimum detectable concentration of 23.4 pg·mL−1. N-terminal pro-brain natriuretic peptide (NT-proBNP) were measured by ELISA (Human NT-proBNP ELISA kit, Cusabio Techology, Wuhan, China) with a detection range of 0.313-20 ng·mL−1, a minimum detectable concentration of 0.216 ng·mL−1

**1.8. Sample size estimation**

Considering ECV quantified by CMR as one of the relevant imaging parameters of RV adaptation and accepting an alpha risk of 0.05 after the Dunnet correction (for M1, M2, and M3 vs. M0) and a beta risk of 0.2, a sample size of 7 animals in each group was considered sufficient to detect a difference equal to or greater than 8 percentage units. A common standard deviation of 5 percentage units was assumed based on previous studies,(2, 4) together with a loss to follow-up rate of 20%. Proteomics analysis was performed with 80 samples obtained at 1 and 8 months post intervention from 16 randomly selected animals (n=4 per group), which was considered adequate for exploratory purposes(22). For the metabolomics study, all samples were used as this technique has greater variability.

**2. SUPPLEMENTARY TABLES**

**Supplementary Table 1**. Stable isotope labeling of plasma samples with TMT-10plex. Labeling with TMT reagents was conducted as indicated on 80 plasma samples from 8 animals subjected to aortopulmonary shunt (M2) or the sham (M0) intervention (TMT 1, TMT 3, TMT 5, and TMT 7) and 8 animals subjected to pulmonary vein banding (M1) or pulmonary artery banding (M3) (TMT 2, TMT 4, TMT 6, and TMT 8).

**Supplementary Table 2.** Primer sequences for quantitative real-time PCR of porcine genes.

**Supplementary Table 3**. Contrast CT angiography measurements at end of follow-up (month 8).

| **Aorto-Pulmonary artery shunt (M2)** | | | |
| --- | --- | --- | --- |
| **Diameter 1 (mm)** | **Diameter 2 (mm)** | **Area (mm^2^)** |  |
| 14.3 (9.7-15.3) | 13.0 (7.8-16.1) | 144.1 (73.0-183.2) | NA |
| **Pulmonary artery banding (M3)** | | | |
| **Diameter 1 (mm)** | **Diameter 2 (mm)** | **Area (mm^2^)** | **% PA stenosis** |
| 9.7 (8.8-10.9) | 9.3 (7.9-10.4) | 70.1 (59.9-84.6) | 83.0 (80.6-88.0) |

**Supplementary Table 4**. Interim hemodynamic and CMR characterization of animal models at 1-month follow-up.

|  | **M1**  **(n=8)** | **M2**  **(n=6)** | **M3**  **(n=10)** | **M0**  **(n=9)** | **P value among groups** |
| --- | --- | --- | --- | --- | --- |
| **Hemodynamic measures** | | | | |  |
| Weight, kg | 22.2 (20.5-23.0) | 25.2 (21.5-35.0) | 24.2 (18.5-28.0) | 27.0 (22.5-28.0) | 0.306 |
| Oxygen saturation, % | 91.5 (89.5-93.5) | 88.0 (85.0-92.0) | 90.0 (89.0-93.0) | 88.5 (83.0-91.5) | 0.736 |
| HR, bpm | 84.0 (76.0-95.5) | 78.5 (70.0-100.0) | 80.0 (78.5-93.5) | 100 (69.0-125.5) | 0.737 |
| SBP, mmHg | 98.5 (92.5-116) | 131 (120-137) | 113 (102-119) | 116 (102-124) | 0.019γ |
| RVSP, mmHg | 40.0 (32.5-42.5) | 39.5 (37.0-65.0) | 50.0 (40.5-51.0) | 29.0 (22.5-36.0) | 0.049 ϕ |
| MPAP, mmHg | 30.5 (25.5-32.5) | 31.0 (27.0-44.0) | 25.0 (20.0-26.0) | 25.5 (24.5-26.0) | 0.140 |
| LVEDP, mmHg | 5.5 (5.0-8.0) | 9.5 (8.0-11.0) | 6.0 (5.0-10.0) | 7.0 (5.0-9.0) | 0.273 |
| CI, L/min/m^2^ | 3.2 (2.3-3.9) | 2.3 (2.0-3.0) | 2.2 (2.1-2.2) | 3.1 (2.2-3.5) | 0.247 |
| iPVR, WU*m^2^ | 6.7 (6.1-8.7) | 11.2 (7.4-13.2) | 7.0 (5.5-9.2) | 6.7 (5.4-9.8) | 0.212 |
| **CMR** | | | | |  |
| RV mass, (g/m^2^ | 18.4 (17.3-20.1) | 18.3 (15.8-19.2) | 17.5 (15.6-21.3) | 16.9 (15.5-17.5) | 0.317 |
| RV end-diastolic volume, mL/m^2^ | 69.5 (65.8-77.4) | 65.2 (51.7-72.5) | 69.9 (62.7-78.9) | 58.2 (54.9-64.2) | 0.154 |
| RV end-systolic volume, mL/m^2^ | 30.3 (27.0-37.7) | 27.6 (21.5-29.9) | 28.3 (25.5-34.5) | 21.7 (19.3-30.0) | 0.079 |
| RV ejection fraction, % | 55.0 (49.5-58.2) | 57.5 (55.0-65.0) | 59.6 (56.0-61.8) | 61.0 (57.0-62.1) | 0.102 |
| RV to LV end-diastolic volume ratio | 0.45 (0.41-0.47) | 0.32 (0.27-0.34) | 0.43 (0.41-0.50) | 0.35 (0.34-0.38) | 0.002ϕ |
| RV to LV end-systolic volume ratio | 1.0 (0.97-1.2) | 0.63 (0.58-0.73) | 1.0 (0.85-1.1) | 0.86 (0.83-0.88) | <0.001γ |
| RV to LV mass ratio | 1.0 (0.86-1.25) | 0.67 (0.60-0.73) | 0.94 (0.83-1.1) | 0.87 (0.74-1.01) | 0.013 |

CI, cardiac index; HR, heart rate; iPVR, indexed pulmonary vascular resistance; LV, left ventricle; LVEDP, left ventricular end-diastolic pressure; MPAP, mean pulmonary arterial pressure; RVSP, right ventricular systolic pressure; SBP, systolic blood pressure; RV, right ventricle. *p<0.05 for the post-hoc comparison M1 (pulmonary vein banding) vs. M0 (Control); γp<0.05 for the post-hoc comparison M2 (aorto-pulmonary shunt) vs. M0 (Control); ϕ p<0.05 for the post-hoc comparison M3 (pulmonary artery banding) vs. M0 (Control).

**Supplementary Table 5**. Statistically significant altered metabolites and the classes to which they belong found in plasma at month 8 in M1 compared to M0 using LC-MS (HILIC and lipidomics). Annotated metabolites are grouped according to subclass classification. Only those that could be annotated based on *m/z* were considered.

| **Group** | **Name** | **Formula** | **Measured mass** | **RT (min)** | ***p*-value (FDR)** | **Change**  **M1 vs M0 (%)** | **Detection method** |
| --- | --- | --- | --- | --- | --- | --- | --- |
| Alkanes | Nonane | C_9_H_2_0 | 150.1369 | 6.0 | 0.04 | -92 | HILIC + |
| Amines | Diethanolamine | C_4_H_11_NO_2_ | 105.0791 | 7.0 | 0.04 | -22 | HILIC + |
| Amino acids, peptides, and analogues | 4-Amino-2-methylidenebutanoate | C_5_H_9_NO_2_ | 115.0634 | 9.2 | 0.04 | -70 | HILIC + |
|  | Aminoheptanoate | C_7_H_15_NO_2_ | 145.1103 | 6.7 | 0.04 | -26 | HILIC + |
|  | Arginine | C_6_H_14_N_4_O_2_ | 174.1122 | 13.1 | 0.04 | -35 | HILIC + |
|  | Dimethylarginine | C_8_H_18_N_4_O_2_ | 202.1432 | 12.1 | 0.04 | -38 | HILIC + |
|  | Guanidinosuccinate | C_5_H_9_N_3_O_4_ | 175.0590 | 11.6 | 0.04 | 74 | HILIC + |
|  | Histidine | C_6_H_9_N_3_O_2_ | 155.0697 | 12.8 | 0.04 | -51 | HILIC + |
|  | Isoleucine | C_6_H_13_NO_2_ | 213.0979 | 4.2 | 0.03 | 21 | HILIC - |
|  | Isoleucylproline_B | C_11_H_20_N_2_O_3_ | 228.1474 | 10.0 | 0.05 | -32 | HILIC + |
|  | n-Trimethyllysine | C_9_H_20_N_2_O_2_ | 188.1529 | 13.0 | 0.04 | -32 | HILIC + |
|  | Phenylacetylglycine | C_10_H_11_NO_3_ | 193.074 | 2.7 | 0.04 | 36 | HILIC - |
|  | Pipecolate | C_6_H_11_NO_2_ | 129.0794 | 13.1 | 0.04 | 41 | HILIC + |
|  | Proline betaine | C_7_H_13_NO_2_ | 121.0829 | 10.1 | 0.04 | -81 | HILIC + |
|  | Tripeptide_1 | C_15_H_26_N_6_O_8_ | 418.1806 | 3.9 | 0.03 | 28 | HILIC - |
| Benzophenones | 2-Amino-5-benzoylbenzimidazole | C_14_H_11_N_3_O | 237.0925 | 2.4 | 0.04 | -72 | HILIC + |
| Carbohydrates and carbohydrate conjugates | Hexose | C_6_H_12_O_6_ | 180.0634 | 5.5 | 0.03 | -28 | HILIC - |
| Fatty acids and conjugates | FA 6:0;O | C_6_H_12_O_3_ | 132.0787 | 2.3 | 0.04 | -35 | HILIC - |
| Glycerophosphocholines | LPC 14:0 | C_22_H_46_NO_7_P | 453.3208 | 4.9 | 0.04 | -27 | HILIC + |
|  | PC 36:7 | C_44_H_74_NO_8_P | 775.5050 | 4.4 | 0.05 | 12 | HILIC + |
| Glycosphingolipids | Ganglioside GA2 (d34:1) | C_54_H_100_N_2_O_18_ | 1086.6638 | 3.5 | 0.05 | 41 | HILIC + |
|  | Ganglioside GA2 (d36:2) | C_56_H_102_N_2_O_18_ | 1112.6773 | 3.5 | 0.04 | 27 | HILIC + |
| Halobenzenes | Ketamine | C_13_H_16_ClNO | 237.0925 | 1.7 | 0.04 | -63 | HILIC + |
| Hybrid peptides | Carnosine | C_9_H_14_N_4_O_3_ | 226.1071 | 13.4 | 0.04 | -21 | HILIC + |
| Indoles | Indolylmethylthiohydroximate | C_10_H_10_N_2_OS | 206.0502 | 2.3 | 0.04 | -35 | HILIC + |
| Indolyl carboxylic acids and derivatives | Indolelactate | C_11_H_11_NO_3_ | 205.0740 | 2.8 | 0.03 | -39 | HILIC - |
| Phenylpropanoic acids | Phenyllactate | C_9_H_10_O_3_ | 166.0627 | 2.2 | 0.03 | -57 | HILIC - |
|  | Hydroxyphenyllactate | C_9_H_10_O_4_ | 182.0581 | 3.1 | 0.03 | -30 | HILIC - |
| Purines and purine derivatives | Hypoxanthine | C_5_H_4_N_4_O | 136.0387 | 3.6 | 0.04 | -25 | HILIC + |
| Ureas | Ureidopropionate | C_4_H_8_N_2_O_3_ | 132.0534 | 5.8 | 0.04 | -35 | HILIC + |

+, positive ionization mode; -, negative ionization mode; FA, fatty acid; FDR, false discovery rate; HILIC, hydrophilic interaction liquid chromatography; LPC, lysophosphatidylcholine; PC, phosphatidylcholine; RT, retention time.

**Supplementary Table 6**. Statistically significant altered metabolites and the classes to which they belong found in plasma at month 8 in M2 compared to M0 using LC-MS (HILIC and lipidomics). Annotated metabolites are grouped according to subclass classification. Only those that could be annotated based on *m/z* were considered.

| **Group** | **Name** | **Formula** | **Measured mass** | **RT (min)** | ***p*-value (FDR)** | **Change M2 vs M0 (%)** | **Detection method** |
| --- | --- | --- | --- | --- | --- | --- | --- |
| Alcohols and polyols | Pantothenate | C_9_H_17_NO_5_ | 219.1106 | 4.5 | 0.032 | -40 | HILIC - |
| Alpha-keto acids and derivatives | Pyruvate | C_3_H_4_O_3_ | 88.0160 | 2.7 | 0.027 | -20 | HILIC - |
| Amines | Diethanolamine | C_4_H_11_NO_2_ | 105.0791 | 7.0 | 0.027 | -25 | HILIC + |
| Amino acids, peptides, and analogues | 4-Amino-2-methylidenebutanoate | C_5_H_9_NO_2_ | 115.0634 | 9.2 | 0.017 | -72 | HILIC + |
|  | Alanine | C_3_H_7_NO_2_ | 89.0480 | 6.7 | 0.013 | -71 | HILIC - |
|  | Alanylisoleucine | C_9_H_18_N_2_O_3_ | 202.1317 | 9.0 | 0.030 | -55 | HILIC + |
|  | Arginine | C_6_H_14_N_4_O_2_ | 174.1118 | 17.0 | 0.038 | -22 | HILIC - & + |
|  | Asparagine | C_4_H_8_N_2_O_3_ | 132.0538 | 10.9 | 0.025 | -15 | HILIC + |
|  | Creatinine | C_4_H_7_N_3_O | 113.0589 | 2.6 | 0.038 | -23 | HILIC - & + |
|  | Cystine | C_6_H_12_N_2_O_4_S_2_ | 240.0238 | 13.2 | 0.021 | -53 | HILIC + |
|  | Dimethylarginine | C_8_H_18_N_4_O_2_ | 202.1432 | 12.1 | 0.017 | -51 | HILIC + |
|  | Guanidinoacetate | C_3_H_7_N_3_O_2_ | 117.0536 | 10.4 | 0.036 | -29 | HILIC + |
|  | Histidine | C_6_H_9_N_3_O_2_ | 155.0697 | 12.8 | 0.019 | -55 | HILIC + |
|  | Homoserine | C_4_H_9_NO_3_ | 119.0582 | 6.8 | 0.032 | -74 | HILIC - |
|  | Isoleucylproline_A | C_11_H_20_N_2_O_3_ | 228.1472 | 9.5 | 0.017 | -48 | HILIC + |
|  | Leucylproline | C_11_H_20_N_2_O_3_ | 228.1474 | 8.3 | 0.028 | -49 | HILIC + |
|  | Methylhistidine_A | C_7_H_11_N_3_O_2_ | 169.0851 | 12.1 | 0.017 | -51 | HILIC + |
|  | Methylhistidine_B | C_7_H_11_N_3_O_2_ | 169.0852 | 13.0 | 0.027 | -43 | HILIC + |
|  | n-Trimethyllysine | C_9_H_20_N_2_O_2_ | 188.1529 | 13.0 | 0.027 | -39 | HILIC + |
|  | Phenylalanine | C_9_H_11_NO_2_ | 165.0791 | 3.7 | 0.007 | -22 | HILIC - |
|  | Proline | C_5_H_9_NO_2_ | 115.0637 | 9.0 | 0.025 | -23 | HILIC + |
|  | Isoleucylproline_B | C_11_H_20_N_2_O_3_ | 228.1474 | 10.0 | 0.017 | -46 | HILIC + |
|  | Pyroglutamate | C_5_H_7_NO_3_ | 129.0426 | 5.4 | 0.042 | -25 | HILIC - |
|  | Threonine | C_4_H_9_NO_3_ | 119.0583 | 6.6 | 0.008 | -34 | HILIC - & + |
|  | Tripeptide_A | C_16_H_31_N_3_O_4_S | 361.2058 | 2.6 | 0.048 | -57 | Lipidomics + |
|  | Tripeptide_B | C_16_H_31_N_3_O_5_ | 345.2261 | 8.3 | 0.030 | -44 | HILIC + |
|  | Tripeptide_C | C_15_H_28_N_6_O_5_ | 372.2125 | 13.4 | 0.027 | -36 | HILIC + |
|  | Tripeptide_D | C_23_H_36_N_6_O_4_ | 460.2798 | 1.9 | 0.015 | -2 | Lipidomics + |
|  | Tyrosine | C_9_H_11_NO_3_ | 181.0741 | 5.1 | 0.003 | -28 | HILIC - & + |
| Aminopyridines and derivatives | Aminopyridine | C_5_H_6_N_2_ | 94.0532 | 13.1 | 0.030 | -78 | HILIC + |
| Aniline and substituted anilines | 1,2-Diaminobenzene | C_6_H_8_N_2_ | 108.0686 | 11.3 | 0.025 | -59 | HILIC + |
| Benzene and substituted derivatives | Mandelate | C_8_H_8_O_3_ | 152.0474 | 1.4 | 0.048 | -46 | HILIC - |
| Bile acids and derivatives | ST 24:2;O4 | C_24_H_38_O_4_ | 390.2750 | 1.9 | 0.042 | 82 | HILIC - |
|  | ST 26:1;O4 | C_26_H_44_O_4_ | 442.2994 | 3.7 | 0.020 | 39 | Lipidomics + |
| Carbohydrates and carbohydrate conjugates | Hexose | C_6_H_12_O_6_ | 180.0634 | 5.5 | 0.042 | -27 | HILIC - |
| Carbonyl compounds | Guanidinobutanal | C_5_H_11_N_3_O | 129.0900 | 5.9 | 0.030 | -24 | HILIC + |
| Ceramides | Cer 38:2;O2 | C_38_H_73_NO_3_ | 651.5793 | 11.7 | 0.045 | 55 | Lipidomics - |
|  | Cer 40:2;O2 | C_40_H_77_NO_3_ | 679.6069 | 12.1 | 0.045 | 70 | Lipidomics - |
| Diradylglycerols | DG 42:0 | C_45_H_88_O_5_ | 725.6927 | 12.6 | 0.037 | 80 | Lipidomics + |
| Drugs | Streptothricin F | C_19_H_34_N_8_O_8_ | 502.2495 | 3.5 | 0.047 | 36 | Lipidomics - |
| Fatty acids and conjugates | Aminooctanoate | C_8_H_17_NO_2_ | 159.1257 | 7.3 | 0.036 | -38 | HILIC + |
|  | FA 5:0;O | C_5_H_10_O_3_ | 118.0631 | 2.6 | 0.003 | -52 | HILIC - |
|  | FA 6:1;O | C_6_H_10_O_3_ | 130.0632 | 2.0 | 0.015 | -26 | HILIC - |
|  | FA 6:0;O | C_6_H_12_O_3_ | 132.0787 | 2.3 | 0.007 | -56 | HILIC - |
|  | FA 10:0;O | C_10_H_20_O_3_ | 188.1415 | 2.2 | 0.048 | 53 | HILIC - |
|  | FA 15:0 | C_15_H_30_O_2_ | 242.2245 | 3.0 | 0.045 | 32 | Lipidomics - |
|  | FA 14:1;O3 | C_14_H_26_O_5_ | 256.1677 | 2.09 | 0.048 | 103 | HILIC - |
|  | FA 14:1;O2 | C_14_H_26_O_4_ | 258.1833 | 2.03 | 0.048 | 87 | HILIC - |
|  | FA 17:1 | C_17_H_32_O_2_ | 268.2403 | 3.19 | 0.048 | 74 | Lipidomics - |
|  | FA 17:0 | C_17_H_34_O_2_ | 270.2558 | 3.9 | 0.045 | 57 | Lipidomics - |
|  | FA 18:3 | C_18_H_30_O_2_ | 278.2247 | 2.5 | 0.048 | 53 | Lipidomics - |
|  | FA 18:2 | C_18_H_30_O_2_ | 280.2409 | 3.04 | 0.045 | 60 | Lipidomics - |
|  | FA 19:1 | C_19_H_36_O_2_ | 296.2713 | 4.03 | 0.047 | 66 | Lipidomics - |
|  | FA 20:4 | C_20_H_32_O_2_ | 304.2402 | 2.94 | 0.045 | 67 | Lipidomics - |
|  | FA 20:3_A | C_20_H_34_O_2_ | 306.2554 | 3.51 | 0.045 | 57 | Lipidomics - |
|  | FA 20:3_B | C_20_H_34_O_2_ | 306.2557 | 3.35 | 0.045 | 57 | Lipidomics - |
|  | FA 20:2 | C_20_H_36_O_2_ | 308.2715 | 3.86 | 0.045 | 61 | Lipidomics - |
|  | FA 22:5 | C_22_H_34_O_2_ | 330.2558 | 3.2 | 0.047 | 42 | Lipidomics - |
|  | FA 22:4_B | C_22_H_36_O_2_ | 332.2716 | 3.7 | 0.045 | 49 | Lipidomics - |
|  | FA 22:4_A | C_22_H_36_O_2_ | 332.2719 | 1.4 | 0.048 | 23 | HILIC - |
|  | FA 20:3;O2 | C_20_H_34_O_4_ | 338.2441 | 1.06 | 0.048 | 58 | Lipidomics - |
| Fatty esters | FAHFA 38:6,O | C_38_H_62_O_4_ | 582.4623 | 3.0 | 0.045 | 136 | Lipidomics - |
|  | FAHFA 46:5,O | C_46_H_82_O_4_ | 698.6216 | 12.5 | 0.045 | 40 | Lipidomics - |
| Glycerophosphates | PA O-37:0 | C_40_H_81_O_7_P | 704.5790 | 4.3 | 0.045 | 39 | Lipidomics - |
| Glycerophosphocholines | PC 37:1 | C_45_H_88_NO_8_P | 801.6237 | 2.4 | 0.032 | 42 | HILIC + |
|  | PC 18:0_20:4 | C_46_H_84_NO_8_P | 809.5941 | 10.2 | 0.027 | 40 | Lipidomics + |
| Glycerophosphoglycerols | PG O-42:4 | C_48_H_89_O_9_P | 862.6209 | 11.6 | 0.048 | 55 | Lipidomics + |
| Glycerophosphoinositols | PI 18:2_16:0 | C_43_H_79_O_13_P | 834.5247 | 6.3 | 0.045 | 70 | Lipidomics - |
|  | PI 18:0_20:4 | C_47_H_83_O_13_P | 886.5581 | 7.5 | 0.010 | 36 | Lipidomics + |
| Glycosphingolipids | Ganglioside GA2 (d34:1) | C_54_H_100_N_2_O_18_ | 1086.6638 | 3.5 | 0.028 | 45 | HILIC + |
| Heteroaromatic compounds | 2,5-Dimethyl-3-furanthiol | C_6_H_8_OS | 188.0499 | 3.8 | 0.008 | -39 | HILIC - |
| Hybrid peptides | Anserine/Homocarnosine | C_10_H_16_N_4_O_3_ | 240.1231 | 11.3 | 0.008 | -37 | HILIC - |
|  | Carnosine | C_9_H_14_N_4_O_3_ | 226.1071 | 13.4 | 0.027 | -26 | HILIC + |
| Indoles | Indoleacrylate | C_11_H_9_NO_2_ | 187.0637 | 7.7 | 0.030 | -28 | HILIC + |
| Indolyl carboxylic acids and derivatives | Indolelactate | C_11_H_11_NO_3_ | 205.0740 | 2.8 | 0.010 | -61 | HILIC - |
|  | Tryptophan | C_11_H_12_N_2_O_2_ | 204.0900 | 4.1 | 0.008 | -37 | HILIC - |
| Organosulfonic acids and derivatives | Isethionate | C_2_H_6_O_4_S | 125.9987 | 2.9 | 0.042 | -30 | HILIC - |
|  | Taurine | C_2_H_7_NO_3_S | 125.0147 | 5.6 | 0.015 | -20 | HILIC - |
| Phenylpropanoic acids | Hydroxyphenyllactate | C_9_H_10_O_4_ | 182.0581 | 3.1 | 0.003 | -53 | HILIC - |
|  | Phenyllactate | C_9_H_10_O_3_ | 166.0627 | 2.2 | 0.032 | -63 | HILIC - |
| Phosphosphingolipids | SM 32:1;O2 | C_37_H_75_N_2_O_6_P | 674.5362 | 6.0 | 0.010 | 49 | Lipidomics + |
|  | SM 34:1;O2 | C_39_H_79_N_2_O_6_P | 702.5677 | 7.4 | 0.010 | 66 | Lipidomics + & - |
|  | SM 38:2;O2 | C_43_H_85_N_2_O_6_P | 756.6141 | 9.9 | 0.010 | 48 | Lipidomics + |
|  | SM 39:1;O2 | C_44_H_89_N_2_O_6_P | 772.6477 | 11.8 | 0.020 | 20 | Lipidomics + |
|  | SM 40:2;O2 | C_45_H_89_N_2_O_6_P | 784.6451 | 11.7 | 0.037 | 46 | Lipidomics + |
|  | SM 41:1;O2 | C_46_H_93_N_2_O_6_P | 800.6773 | 12.1 | 0.010 | 49 | Lipidomics + & - |
|  | SM 42:1;O2 | C_47_H_95_N_2_O_6_P | 814.6931 | 12.3 | 0.020 | 54 | Lipidomics + |
|  | SM 43:1;O2 | C_48_H_97_N_2_O_6_P | 828.7116 | 12.4 | 0.005 | 14 | Lipidomics + |
| Purine nucleosides | 2-Aminoadenosine | C_10_H_14_N_6_O_4_ | 282.1082 | 2.0 | 0.042 | -52 | HILIC - |
|  | Guanosine | C_10_H_13_N_5_O_5_ | 283.0916 | 7.3 | 0.036 | -47 | HILIC + |
|  | Inosine | C_10_H_12_N_4_O_5_ | 268.0818 | 5.8 | 0.036 | -71 | HILIC + |
|  | Xanthosine | C_10_H_12_N_4_O_6_ | 284.0758 | 4.9 | 0.042 | -31 | HILIC - |
| Purines and purine derivatives | Guanine | C_5_H_5_N_5_O | 151.0496 | 5.6 | 0.025 | -27 | HILIC + |
|  | Hypoxanthine | C_5_H_4_N_4_O | 136.0387 | 2.5 | 0.010 | -34 | HILIC - & + |
|  | Uric acid | C_5_H_4_N_4_O_3_ | 168.0284 | 5.1 | 0.015 | -30 | HILIC - |
|  | Xanthine | C_5_H_4_N_4_O_2_ | 152.0333 | 2.6 | 0.010 | -38 | HILIC - |
| Pyrazolo[3,4-d]pyrimidines | Allopurinol | C_5_H_4_N_4_O | 136.0386 | 5.8 | 0.036 | -68 | HILIC + |
| Pyrimidine 2'-deoxyribonucleosides | 5-Methyldeoxycytidine | C_10_H_15_N_3_O_4_ | 241.1058 | 5.3 | 0.028 | -28 | HILIC + |
| Pyrimidine nucleosides | Methylcytidine | C_10_H_15_N_3_O_5_ | 257.1025 | 11.1 | 0.036 | -24 | HILIC + |
| Quaternary ammonium salts | Neurine | C_5_H_13_NO | 103.1000 | 6.4 | 0.032 | -36 | HILIC + |
| Short-chain keto acids and derivatives | Ketoisovalerate | C_5_H_8_O_3_ | 116.0474 | 2.1 | 0.008 | -20 | HILIC - |
| Sphingoid bases | Sphingosine-1-phosphate | C_18_H_38_NO_5_P | 379.2473 | 6.7 | 0.028 | -22 | HILIC + |
| Sulfonic acids | Hypotaurine | C_2_H_7_NO_2_S | 109.0198 | 10.5 | 0.032 | -31 | HILIC + |
| Triradylglycerols | TG 60:5 | C_63_H_112_O_6_ | 988.8220 | 13.8 | 0.015 | 46 | Lipidomics + |

-, negative ionization mode; +, positive ionization mode; Cer, ceramide, DG, diglyceride; FA, fatty acid; FAHFA, fatty acid esters of hydroxy fatty acids; FDR, false discovery rate; HILIC, hydrophilic interaction liquid chromatography; LPC, lysophosphatidylcholine; PA, phosphatidic acid; PC, phosphatidylcholine; PG, phosphatidylglycerol; PI, phosphatidylinositol; RT, retention time; SM, sphingomyelin; ST, sterol lipid; TG, triglyceride. A, B, and C indicate different isomers,

**Supplementary Table 7**. Statistically significant altered metabolites and the classes to which they belong found in plasma at month 8 in M3 compared to M0 using LC-MS (HILIC and lipidomics). Annotated metabolites are grouped according to subclass classification. Only those that could be annotated based on *m/z* were considered.

| **Group** | **Name** | **Formula** | **Measured mass** | **RT (min)** | ***p*-value (FDR)** | **Change M3 vs M0 (%)** | **Detection method** |
| --- | --- | --- | --- | --- | --- | --- | --- |
| Alpha hydroxy acids and derivatives | Lactiate | C_3_H_6_O_3_ | 90.0319 | 3.8 | 0.042 | 24 | HILIC - |
| Amino acids, peptides, and analogues | Aminooctanoate | C_8_H_17_NO_2_ | 159.1257 | 7.3 | 0.044 | -35 | HILIC + |
|  | Asparagine | C_4_H_8_N_2_O_3_ | 132.0538 | 10.9 | 0.044 | -10 | HILIC + |
|  | Glutamylarginine | C_11_H_21_N_5_O_5_ | 303.1545 | 13.5 | 0.044 | -24 | HILIC + |
|  | N-Methyllysine | C_7_H_16_N_2_O_2_ | 160.1214 | 17.6 | 0.042 | 184 | HILIC - |
|  | Proline | C_5_H_9_NO_2_ | 115.0637 | 9.0 | 0.044 | -16 | HILIC + |
|  | Threonine | C_4_H_9_NO_3_ | 119.0579 | 10.3 | 0.044 | -19 | HILIC + |
|  | Tyrosine | C_9_H_11_NO_3_ | 181.0739 | 8.7 | 0.044 | -24 | HILIC + |
| Bile acids and derivatives | ST 26:1;O4 | C_26_H_44_O_4_ | 442.2991 | 7.6 | 0.002 | 47 | Lipidomics + |
| Carbohydrates and carbohydrate conjugates | Dopamine glucuronide | C_14_H_19_NO_8_ | 329.1142 | 4.5 | 0.042 | -30 | HILIC - |
| Ceramides | Cer 34:1;O2 | C_34_H_67_NO_3_ | 537.5071 | 1.3 | 0.044 | 34 | HILIC + |
|  | Cer 38:1;O2 | C_38_H_75_NO_3_ | 629.5451 | 12.0 | 0.035 | 40 | Lipidomics - |
|  | Cer 42:2; O2 | C_42_H_81_NO_3_ | 647.6206 | 12.3 | 0.040 | 43 | Lipidomics - |
|  | Cer 40:2;O2 | C_40_H_77_NO_3_ | 679.6069 | 12.1 | 0.036 | 35 | Lipidomics - |
| Diradylglycerols | DG 40:0 | C_43_H_84_O_5_ | 679.6580 | 12.3 | 0.040 | 55 | Lipidomics + |
|  | DG 42:0 | C_45_H_88_O_5_ | 725.6927 | 12.6 | 0.005 | 65 | Lipidomics + |
|  | DG 42:2 | C_45_H_84_O_5_ | 703.6466 | 12.1 | 0.033 | 30 | Lipidomics + |
|  | DG 46:1 | C_45_H_86_O_5_ | 705.6751 | 12.3 | 0.015 | 38 | Lipidomics + |
| Fatty esters | FAHFA 37:1;O | C_37_H_70_O_4_ | 595.5643 | 9.5 | 0.015 | 26 | Lipidomics + |
|  | FAHFA 46:5;O | C_46_H_82_O_4_ | 698.6216 | 12.5 | 0.026 | 37 | Lipidomics - |
| Glycerophosphocholines | LPC 17:0 | C_25_H_52_NO_7_P | 509.3486 | 3.1 | 0.033 | 34 | Lipidomics + & HILIC + |
|  | PC 16:0_18:2 | C_42_H_80_NO_8_P | 757.5621 | 8.1 | 0.001 | 58 | Lipidomics + |
|  | PC 18:0_18:1 | C_44_H_86_NO_8_P | 787.6097 | 11.6 | 0.033 | 37 | Lipidomics + |
|  | PC 18:0_20:3_A | C_46_H_86_NO_8_P | 811.6092 | 11.4 | 0.033 | 46 | Lipidomics + |
|  | PC 18:0_20:3_B | C_46_H_86_NO_8_P | 811.6104 | 11.5 | 0.005 | 62 | Lipidomics + |
|  | PC 18:1_18:2 | C_44_H_82_NO_8_P | 843.5980 | 8.9 | 0.036 | 45 | Lipidomics - |
|  | PC 37:1 | C_45_H_88_NO_8_P | 801.6237 | 2.4 | 0.044 | 58 | HILIC + |
|  | PC 40:3 | C_48_H_90_NO_8_P | 839.6456 | 11.9 | 0.004 | 64 | Lipidomics + |
|  | PC 40:4 | C_48_H_88_NO_8_P | 837.6193 | 1.6 | 0.044 | 35 | HILIC + |
|  | PC O-34:3 | C_42_H_80_NO_7_P | 741.5674 | 9.2 | 0.010 | 24 | Lipidomics + |
|  | PC O-36:1 | C_44_H_88_NO_7_P | 773.6307 | 11.9 | 0.040 | 18 | Lipidomics + |
|  | PC O-36:3_A | C_44_H_84_NO_7_P | 769.5988 | 11.5 | 0.033 | 27 | Lipidomics + |
|  | PC O-36:3_B | C_44_H_84_NO_7_P | 769.5998 | 9.8 | 0.008 | 33 | Lipidomics + |
| Glycerophosphoethanolamines | PE 40:1 | C_45_H_88_NO_8_P | 801.6215 | 11.9 | 0.005 | 57 | Lipidomics + |
|  | PE 42:5 | C_47_H_84_NO_8_P | 821.5928 | 9.2 | 0.048 | 33 | Lipidomics + |
| Glycerophosphoinositols | PI 18:0_20:4 | C_47_H_83_O_13_P | 886.5581 | 7.5 | 0.033 | 21 | Lipidomics + |
|  | PI 36:1 | C_45_H_85_O_13_P | 886.5563 | 3.8 | 0.044 | 31 | HILIC + |
| Neutral glycosphingolipids | HexCer 42:2;O2 | C_48_H_91_NO_8_ | 869.6925 | 12.0 | 0.035 | 32 | Lipidomics - |
| Phosphosphingolipids | SM 34:2;O2 | C_39_H_77_N_2_O_6_P | 700.5519 | 1.6 | 0.044 | -21 | HILIC + |
|  | SM 35:1;O2 | C_40_H_81_N_2_O_6_P | 716.5818 | 8.3 | 0.033 | 34 | Lipidomics + |
| Polyprenols | Undecaprenyl phosphate mannose | C_61_H_101_O_9_P | 1030.6968 | 4.1 | 0.044 | 29 | HILIC + |
| Purine nucleosides | N,N-Dimethyladenosine | C_12_H_17_N_5_O_4_ | 295.1267 | 9.0 | 0.044 | -26 | HILIC + |
| Sterols | ST 29:2;O3 | C_29_H_48_O_3_ | 444.3674 | 11.5 | 0.015 | 42 | Lipidomics + |
| Triradylglycerols | TG 54:4 | C_57_H_102_O_6_ | 920.7374 | 13.5 | 0.019 | 53 | Lipidomics + |
|  | TG 56:2 | C_59_H_110_O_6_ | 938.8050 | 14.1 | 0.040 | 47 | Lipidomics + |
|  | TG 56:5 | C_59_H_104_O_6_ | 925.8119 | 14.7 | 0.003 | 91 | Lipidomics + |
|  | TG 56:6 | C_59_H_102_O_6_ | 923.7956 | 14.1 | 0.023 | 39 | Lipidomics + |
|  | TG 58:2 | C_61_H_114_O_6_ | 966.8368 | 14.7 | 0.004 | 88 | Lipidomics + |
|  | TG 60:5 | C_63_H_112_O_6_ | 988.8220 | 13.8 | 0.006 | 48 | Lipidomics + |
|  | TG 60:6 | C_63_H_110_O_6_ | 986.8058 | 13.5 | 0.023 | 39 | Lipidomics + |
|  | TG 60:7 | C_63_H_108_O_6_ | 984.7906 | 13.2 | 0.012 | 46 | Lipidomics + |
|  | TG 62:6 | C_65_H_114_O_6_ | 1007.8907 | 14.7 | 0.005 | 84 | Lipidomics + |
|  | TG 62:8 | C_65_H_110_O_6_ | 1003.8609 | 13.7 | 0.028 | 36 | Lipidomics + |

-, negative ionization mode; +, positive ionization mode; Cer, ceramide, DG, diglyceride; FAHFA, fatty acid esters of hydroxy fatty acids; FDR, false discovery rate; HexCer, hexosylceramide; HILIC, hydrophilic interaction liquid chromatography; LPC, lysophosphatidylcholine; PC, phosphatidylcholine; PE, phosphatidylethanolamines; PI, phosphatidylinositol; RT, retention time; SM, sphingomyelin; ST, sterol lipid; TG, triglyceride. A, B, and C indicate different isomers,

**Supplementary Table 8**. Relative plasma protein abundance in the different pig models. Only those proteins identified at 1% FDR with more than one peptide were considered (for details see attached Excel file).

**Supplementary table 9**. Significant functional categories revealed by the enrichment analysis carried out with the proteins found altered in the comparative proteomics analyses. A p-value cutoff of 0.1 was used as the criterion for protein change (for details see attached Excel file).

**Supplementary Table 10**. Pearson correlation analysis between quantitative data from analysis by omics (proteins, metabolites, and lipids) and hemodynamics (iPVR and MPAP), and imaging (iRVmass, iRVESV, RVEF and ECV).

| Qi | Qj | pearson coefficient | p-value |
| --- | --- | --- | --- |
| iRV mass | 2-Amino-5-benzoylbenzimidazole | -0,306629794 | 1,08E-02 |
| ECV RV AIP | 3-Methylhistidine | -0,462745988 | 2,13E-04 |
| ECV RV IIP | 3-Methylhistidine | -0,667739905 | 1,74E-08 |
| ECV SIV | 3-Methylhistidine | -0,536063171 | 1,03E-05 |
| iPVR | 3-Methylhistidine | -0,386038466 | 1,65E-03 |
| iRVESV | 3-Methylhistidine | -0,409092022 | 7,15E-04 |
| MPAP | 3-Methylhistidine | -0,503028857 | 2,86E-05 |
| RVEF | 3-Methylhistidine | 0,556624993 | 2,85E-06 |
| iRV mass | 3-Methylhistidine | -0,413537775 | 7,68E-04 |
| ECV RV IIP | 1-Methyl-L-histidine | -0,562964832 | 4,68E-06 |
| iPVR | 1-Methyl-L-histidine | -0,59061939 | 8,27E-07 |
| iRVESV | 1-Methyl-L-histidine | -0,47465556 | 8,35E-05 |
| MPAP | 1-Methyl-L-histidine | -0,433246342 | 3,41E-04 |
| RVEF | 1-Methyl-L-histidine | 0,584694119 | 7,20E-07 |
| iRV mass | 1-Methyl-L-histidine | -0,380140525 | 1,93E-03 |
| ECV RV IIP | 2-Aminoadenosine | -0,473935221 | 1,47E-04 |
| iPVR | 2-Aminoadenosine | -0,482230243 | 8,38E-05 |
| iRVESV | 2-Aminoadenosine | -0,376606425 | 1,79E-03 |
| MPAP | 2-Aminoadenosine | -0,342934387 | 4,20E-03 |
| RVEF | 2-Aminoadenosine | 0,678150656 | 2,48E-09 |
| iRV mass | 2-Aminoadenosine | -0,263306528 | 2,50E-02 |
| ECV RV AIP | 4-Guanidinobutanal | -0,409504713 | 1,05E-03 |
| ECV RV IIP | 4-Guanidinobutanal | -0,431588257 | 5,60E-04 |
| ECV SIV | 4-Guanidinobutanal | -0,353909142 | 3,73E-03 |
| iPVR | 4-Guanidinobutanal | -0,349535541 | 4,14E-03 |
| MPAP | 4-Guanidinobutanal | -0,39987795 | 9,36E-04 |
| ECV RV IIP | 6-Methylthioguanine | -0,355283559 | 4,19E-03 |
| iPVR | 6-Methylthioguanine | -0,589017639 | 8,97E-07 |
| iRVESV | 6-Methylthioguanine | -0,581876723 | 8,31E-07 |
| MPAP | 6-Methylthioguanine | -0,524649855 | 1,18E-05 |
| RVEF | 6-Methylthioguanine | 0,711469319 | 1,93E-10 |
| iRV mass | 6-Methylthioguanine | -0,354272796 | 3,69E-03 |
| iPVR | A2M | 0,476505058 | 7,80E-05 |
| iRVESV | A2M | 0,470306772 | 9,76E-05 |
| MPAP | A2M | 0,445344844 | 2,30E-04 |
| iRV mass | A2M | 0,569823794 | 1,52E-06 |
| iRV mass | Ala Ile | -0,375150238 | 2,19E-03 |
| ECV RV IIP | Alanine | -0,56934218 | 3,52E-06 |
| ECV SIV | Alanine | -0,346345734 | 4,46E-03 |
| iPVR | Alanine | -0,465165653 | 1,52E-04 |
| iRVESV | Alanine | -0,445775776 | 2,27E-04 |
| MPAP | Alanine | -0,535311816 | 7,47E-06 |
| RVEF | Alanine | 0,663262119 | 7,00E-09 |
| iRV mass | Alanine | -0,396405635 | 1,25E-03 |
| iRVESV | APOA2 | -0,362716846 | 2,57E-03 |
| iRV mass | APOA2 | -0,389356522 | 1,26E-03 |
| ECV SIV | APOC3 | -0,350565126 | 4,04E-03 |
| RVEF | APOC3 | -0,324807913 | 6,43E-03 |
| ECV SIV | APOH | -0,337376398 | 5,50E-03 |
| ECV RV IIP | APOR | -0,377261863 | 2,08E-03 |
| ECV RV AIP | Arg Thr Pro | -0,384024118 | 2,07E-03 |
| ECV RV IIP | Arg Thr Pro | -0,467401085 | 1,83E-04 |
| ECV SIV | Arg Thr Pro | -0,362580896 | 3,01E-03 |
| MPAP | Arg Thr Pro | -0,383884481 | 1,47E-03 |
| RVEF | Arg Thr Pro | 0,528768659 | 9,91E-06 |
| ECV RV AIP | Arginine | -0,44484699 | 3,75E-04 |
| ECV RV IIP | Arginine | -0,688390907 | 4,39E-09 |
| ECV SIV | Arginine | -0,43889753 | 3,57E-04 |
| iPVR | Arginine | -0,432930016 | 4,30E-04 |
| iRVESV | Arginine | -0,348598526 | 3,66E-03 |
| MPAP | Arginine | -0,499100677 | 3,33E-05 |
| RVEF | Arginine | 0,618858105 | 1,12E-07 |
| iRV mass | Arginine | -0,269853619 | 2,21E-02 |
| ECV RV IIP | C1QC | 0,372859569 | 2,33E-03 |
| RVEF | C1QC | 0,320995091 | 7,01E-03 |
| ECV SIV | C3 | 0,424217534 | 5,60E-04 |
| iPVR | C3 | 0,353370853 | 3,25E-03 |
| iRVESV | C3 | 0,332976683 | 5,32E-03 |
| MPAP | C3 | 0,330505805 | 5,64E-03 |
| iRV mass | C3 | 0,386284769 | 1,37E-03 |
| ECV RV AIP | C4B | 0,450922826 | 2,44E-04 |
| iRV mass | C4B | 0,417094439 | 5,63E-04 |
| ECV RV AIP | C4BPA | -0,362698166 | 3,01E-03 |
| ECV RV IIP | C4BPA | -0,396872757 | 1,23E-03 |
| iRV mass | C4BPB | 0,291462247 | 1,32E-02 |
| ECV RV IIP | C5 | 0,318052621 | 8,45E-03 |
| ECV SIV | C5 | 0,349125214 | 4,18E-03 |
| iRV mass | C5 | 0,339735685 | 4,54E-03 |
| ECV RV AIP | C7 | 0,475087701 | 1,08E-04 |
| ECV RV IIP | C7 | 0,537237496 | 9,81E-06 |
| ECV SIV | C7 | 0,328718635 | 6,69E-03 |
| iPVR | C7 | 0,401141698 | 9,02E-04 |
| iRVESV | C7 | 0,364180704 | 2,47E-03 |
| MPAP | C7 | 0,417448511 | 5,57E-04 |
| iRV mass | C7 | 0,497366658 | 3,56E-05 |
| ECV RV IIP | C8A | 0,314219721 | 9,18E-03 |
| ECV SIV | C8A | 0,675012269 | 5,80E-09 |
| MPAP | C8B | -0,308955646 | 9,14E-03 |
| iRV mass | C8B | -0,265383873 | 2,20E-02 |
| RVEF | Carnosine | 0,30730795 | 9,47E-03 |
| MPAP | Carnosine | -0,3546062 | 3,16E-03 |
| ECV RV AIP | Carnosine | -0,467154623 | 1,85E-04 |
| ECV SIV | Carnosine | -0,516943948 | 2,26E-05 |
| ECV RV IIP | Cer 38:1;O2 | 0,334470635 | 6,72E-03 |
| RVEF | Cer 38:1;O2 | -0,589798975 | 5,53E-07 |
| ECV RV IIP | Cer 38:2;O2 | 0,485820868 | 9,80E-05 |
| RVEF | Cer 38:2;O2 | -0,582001528 | 8,26E-07 |
| ECV RV IIP | Cer 40:0;O3 | 0,345512715 | 5,25E-03 |
| RVEF | Cer 40:0;O3 | -0,525588557 | 1,13E-05 |
| ECV RV IIP | Cer 40:2;O2 | 0,535294256 | 1,52E-05 |
| RVEF | Cer 40:2;O2 | -0,520463732 | 1,41E-05 |
| RVEF | Cer 42:1;O2 | -0,494511033 | 3,98E-05 |
| ECV RV AIP | Cer 44:0;O4 | -0,350504273 | 4,68E-03 |
| RVEF | Cer 44:0;O4 | -0,40478184 | 8,12E-04 |
| ECV RV IIP | Cer 44:1;O2 | 0,366325609 | 3,22E-03 |
| RVEF | Cer 44:1;O2 | -0,46577147 | 1,15E-04 |
| ECV RV IIP | Cer 44:1;O5 | 0,354434763 | 4,27E-03 |
| RVEF | Cer 44:1;O5 | -0,525623608 | 1,13E-05 |
| RVEF | Cer(d14:1/20:0) | -0,371733941 | 2,03E-03 |
| ECV RV IIP | Cer(d18:1/24:1) | 0,365173308 | 3,31E-03 |
| RVEF | Cer(d18:1/24:1) | -0,513963759 | 1,84E-05 |
| ECV RV IIP | Cer(d18:1/26:1) | 0,491384145 | 8,06E-05 |
| RVEF | Cer(d18:1/26:1) | -0,531592356 | 8,78E-06 |
| ECV RV IIP | CFB | 0,343580661 | 4,76E-03 |
| ECV SIV | CFB | 0,458892065 | 1,87E-04 |
| iPVR | CFB | 0,320273236 | 7,12E-03 |
| iRV mass | CFB | 0,284219135 | 1,53E-02 |
| ECV RV AIP | CFH | 0,316584583 | 8,72E-03 |
| ECV SIV | CFH | 0,458657871 | 1,89E-04 |
| ECV SIV | CFI | 0,323846384 | 7,45E-03 |
| iRV mass | COL1A1 | -0,240288546 | 3,46E-02 |
| RVEF | CPB2 | -0,318661424 | 7,39E-03 |
| iRV mass | Cystine | -0,306629794 | 0,010765881 |
| ECV RV AIP | D-Arginine | -0,44892382 | 3,31E-04 |
| ECV RV IIP | D-Arginine | -0,644578042 | 7,20E-08 |
| ECV SIV | D-Arginine | -0,443914179 | 3,05E-04 |
| iPVR | D-Arginine | -0,446887747 | 2,77E-04 |
| iRVESV | D-Arginine | -0,421890705 | 4,86E-04 |
| MPAP | D-Arginine | -0,521366256 | 1,36E-05 |
| RVEF | D-Arginine | 0,62776085 | 6,66E-08 |
| iRV mass | D-Arginine | -0,356452107 | 3,50E-03 |
| ECV RV IIP | ECM1 | -0,310749681 | 9,88E-03 |
| ECV RV AIP | F10 | -0,650555746 | 2,86E-08 |
| ECV RV IIP | F10 | -0,388062889 | 1,56E-03 |
| MPAP | F10 | -0,310074813 | 8,92E-03 |
| iRV mass | F10 | -0,325926623 | 6,27E-03 |
| ECV RV AIP | F11 | 0,509897336 | 2,98E-05 |
| ECV RV IIP | F11 | 0,377935799 | 2,04E-03 |
| iRV mass | F12 | 0,219619931 | 4,88E-02 |
| iPVR | F13B | 0,318321663 | 7,44E-03 |
| iRV mass | F5 | -0,25949215 | 2,46E-02 |
| RVEF | F9 | -0,531000066 | 9,01E-06 |
| RVEF | FA 10:0;O | -0,490269367 | 4,68E-05 |
| iPVR | FA 14:1;O2 | 0,332480662 | 6,15E-03 |
| ECV RV AIP | FA 14:1;O2 | -0,381964237 | 2,19E-03 |
| RVEF | FA 14:1;O2 | -0,537783095 | 6,70E-06 |
| iRV mass | FA 14:1;O2 | 0,23284906 | 4,21E-02 |
| iPVR | FA 14:1;O3 | 0,321454925 | 7,85E-03 |
| RVEF | FA 14:1;O3 | -0,509032149 | 2,25E-05 |
| ECV RV IIP | FA 15:0 | 0,325251269 | 8,20E-03 |
| iPVR | FA 15:0 | 0,485035216 | 7,58E-05 |
| iRVESV | FA 15:0 | 0,349538203 | 3,58E-03 |
| MPAP | FA 15:0 | 0,398883004 | 9,63E-04 |
| RVEF | FA 15:0 | -0,635919857 | 4,07E-08 |
| ECV RV IIP | FA 16:0 | 0,341950375 | 5,69E-03 |
| RVEF | FA 16:0 | -0,397740946 | 9,95E-04 |
| ECV RV IIP | FA 16:1 | 0,346085219 | 5,18E-03 |
| RVEF | FA 16:1 | -0,446001018 | 2,25E-04 |
| iRV mass | FA 16:1 | 0,2229742 | 4,93E-02 |
| ECV RV IIP | FA 16:1;O | 0,365002953 | 3,33E-03 |
| iPVR | FA 16:1;O | 0,478773092 | 9,47E-05 |
| MPAP | FA 16:1;O | 0,314896365 | 8,03E-03 |
| RVEF | FA 16:1;O | -0,614843718 | 1,41E-07 |
| ECV RV IIP | FA 17:0 | 0,420703101 | 7,69E-04 |
| iPVR | FA 17:0 | 0,430754665 | 4,60E-04 |
| iRVESV | FA 17:0 | 0,366843005 | 2,31E-03 |
| MPAP | FA 17:0 | 0,41082436 | 6,79E-04 |
| RVEF | FA 17:0 | -0,61281465 | 1,58E-07 |
| iPVR | FA 17:1 | 0,391875788 | 1,41E-03 |
| iRVESV | FA 17:1 | 0,353192724 | 3,27E-03 |
| MPAP | FA 17:1 | 0,395958903 | 1,05E-03 |
| RVEF | FA 17:1 | -0,614110977 | 1,47E-07 |
| ECV RV IIP | FA 18:2 | 0,337399766 | 6,30E-03 |
| iPVR | FA 18:2 | 0,406190294 | 9,48E-04 |
| iRVESV | FA 18:2 | 0,396579241 | 1,03E-03 |
| MPAP | FA 18:2 | 0,432256783 | 3,51E-04 |
| RVEF | FA 18:2 | -0,647301126 | 2,00E-08 |
| iPVR | FA 18:3 | 0,403443453 | 1,02E-03 |
| iRVESV | FA 18:3 | 0,374868885 | 1,87E-03 |
| MPAP | FA 18:3 | 0,406939698 | 7,62E-04 |
| ECV RV AIP | FA 18:3 | -0,355265355 | 4,19E-03 |
| RVEF | FA 18:3 | -0,642167621 | 2,77E-08 |
| ECV RV IIP | FA 19:1 | 0,352795741 | 4,44E-03 |
| iPVR | FA 19:1 | 0,33779503 | 5,45E-03 |
| iRVESV | FA 19:1 | 0,328562252 | 5,90E-03 |
| MPAP | FA 19:1 | 0,387375401 | 1,33E-03 |
| RVEF | FA 19:1 | -0,551363488 | 3,64E-06 |
| ECV RV IIP | FA 20:2 | 0,434767345 | 5,10E-04 |
| iPVR | FA 20:2 | 0,363951927 | 2,91E-03 |
| iRVESV | FA 20:2 | 0,367803453 | 2,25E-03 |
| MPAP | FA 20:2 | 0,411797443 | 6,60E-04 |
| RVEF | FA 20:2 | -0,617689687 | 1,20E-07 |
| ECV RV IIP | FA 20:3 | 0,430248135 | 5,83E-04 |
| iPVR | FA 20:3 | 0,497614953 | 4,77E-05 |
| iRVESV | FA 20:3 | 0,30605925 | 9,73E-03 |
| MPAP | FA 20:3 | 0,376339658 | 1,80E-03 |
| RVEF | FA 20:3 | -0,686757766 | 1,32E-09 |
| ECV RV IIP | FA 20:4 | 0,409748936 | 1,05E-03 |
| iPVR | FA 20:4 | 0,465245983 | 1,51E-04 |
| iRVESV | FA 20:4 | 0,383691043 | 1,47E-03 |
| MPAP | FA 20:4 | 0,406338887 | 7,75E-04 |
| RVEF | FA 20:4 | -0,673217541 | 3,52E-09 |
| ECV RV IIP | FA 22:4 | 0,40518589 | 1,19E-03 |
| iPVR | FA 22:4 | 0,370861874 | 2,45E-03 |
| iRVESV | FA 22:4 | 0,317133819 | 7,64E-03 |
| MPAP | FA 22:4 | 0,372951428 | 1,97E-03 |
| RVEF | FA 22:4 | -0,573783144 | 1,25E-06 |
| ECV RV IIP | FA 22:5 | 0,42002382 | 7,84E-04 |
| iPVR | FA 22:5 | 0,385476513 | 1,67E-03 |
| iRVESV | FA 22:5 | 0,321504358 | 6,93E-03 |
| MPAP | FA 22:5 | 0,370861065 | 2,08E-03 |
| RVEF | FA 22:5 | -0,643912754 | 2,48E-08 |
| ECV RV IIP | FA 22:5;O4 | 0,365121266 | 3,32E-03 |
| iPVR | FA 22:5;O4 | 0,503360914 | 3,84E-05 |
| iRVESV | FA 22:5;O4 | 0,438769903 | 2,85E-04 |
| MPAP | FA 22:5;O4 | 0,458260872 | 1,49E-04 |
| RVEF | FA 22:5;O4 | -0,742873868 | 1,22E-11 |
| RVEF | FA 5:0;O | 0,622327613 | 9,18E-08 |
| ECV RV IIP | FA 5:0;O | -0,413540383 | 9,41E-04 |
| ECV SIV | FA 5:0;O | -0,447273867 | 2,74E-04 |
| iPVR | FA 5:0;O | -0,562431075 | 3,22E-06 |
| iRVESV | FA 5:0;O | -0,440350659 | 2,71E-04 |
| MPAP | FA 5:0;O | -0,643906634 | 2,48E-08 |
| iRV mass | FA 5:0;O | -0,463873863 | 1,59E-04 |
| RVEF | FA 6:0;O | 0,727999892 | 4,74E-11 |
| ECV RV IIP | FA 6:0;O | -0,419505762 | 7,95E-04 |
| ECV SIV | FA 6:0;O | -0,310244359 | 9,98E-03 |
| iPVR | FA 6:0;O | -0,561428574 | 3,37E-06 |
| iRVESV | FA 6:0;O | -0,392808435 | 1,14E-03 |
| MPAP | FA 6:0;O | -0,600372493 | 3,15E-07 |
| iRV mass | FA 6:0;O | -0,36363319 | 2,94E-03 |
| RVEF | FA 6:1;O | 0,707210531 | 2,73E-10 |
| ECV RV IIP | FA 6:1;O | -0,504636199 | 4,99E-05 |
| iPVR | FA 6:1;O | -0,522809577 | 1,79E-05 |
| MPAP | FA 6:1;O | -0,375886683 | 1,82E-03 |
| ECV RV IIP | FAHFA | 0,360575394 | 3,70E-03 |
| RVEF | FAHFA | -0,466330819 | 1,12E-04 |
| ECV RV IIP | FAHFA 18:2_20:4 | 0,359326288 | 3,81E-03 |
| iPVR | FAHFA 18:2_20:4 | 0,492626074 | 5,74E-05 |
| iRVESV | FAHFA 18:2_20:4 | 0,380324186 | 1,62E-03 |
| MPAP | FAHFA 18:2_20:4 | 0,42097444 | 5,00E-04 |
| RVEF | FAHFA 18:2_20:4 | -0,725501396 | 5,90E-11 |
| ECV RV IIP | FAHFA 42:1O | 0,334293446 | 6,74E-03 |
| RVEF | FAHFA 42:1O | -0,537410208 | 6,81E-06 |
| ECV RV IIP | FAHFA 44:3O | 0,315823338 | 1,00E-02 |
| RVEF | FAHFA 44:3O | -0,513079735 | 1,91E-05 |
| ECV RV IIP | FAHFA 46:5O | 0,354021959 | 4,32E-03 |
| RVEF | FAHFA 46:5O | -0,500365168 | 3,17E-05 |
| ECV RV IIP | FAHFA 46:6O | 0,360479103 | 3,71E-03 |
| RVEF | FAHFA 46:6O | -0,531972751 | 8,64E-06 |
| ECV RV AIP | FCN1 | -0,373758796 | 2,27E-03 |
| ECV RV IIP | FCN1 | -0,349975134 | 4,10E-03 |
| iRV mass | FCN2 | 0,279358241 | 1,68E-02 |
| iPVR | FGB | 0,310979374 | 8,75E-03 |
| iRV mass | FGB | 0,252771164 | 2,78E-02 |
| iRV mass | FGG | 0,233539178 | 3,88E-02 |
| ECV RV AIP | Glutamylarginine | -0,431557583 | 5,61E-04 |
| ECV SIV | Glutamylarginine | -0,481686564 | 8,54E-05 |
| iRV mass | Glycylglycine | -0,406086338 | 9,50E-04 |
| iRVESV | GPX3 | 0,441449075 | 2,61E-04 |
| iRV mass | GPX3 | 0,306829966 | 9,57E-03 |
| ECV RV IIP | Guanidinoacetic acid | -0,536504631 | 1,45E-05 |
| ECV SIV | Guanidinoacetic acid | -0,43955097 | 3,50E-04 |
| ECV RV AIP | Guanidinosuccinic acid | 0,386027115 | 1,97E-03 |
| iPVR | Guanidinosuccinic acid | -0,449255963 | 2,57E-04 |
| RVEF | Guanidinosuccinic acid | 0,315663512 | 7,89E-03 |
| ECV RV IIP | Guanine | -0,568277149 | 3,69E-06 |
| ECV SIV | Guanine | -0,364233678 | 2,89E-03 |
| iPVR | Guanine | -0,474457752 | 1,10E-04 |
| iRVESV | Guanine | -0,39384124 | 1,11E-03 |
| MPAP | Guanine | -0,513976276 | 1,84E-05 |
| RVEF | Guanine | 0,629415102 | 6,04E-08 |
| iRV mass | Guanine | -0,343026532 | 4,82E-03 |
| ECV RV AIP | Guanosine | -0,397244268 | 1,47E-03 |
| ECV RV IIP | Guanosine | -0,594554694 | 1,07E-06 |
| ECV SIV | Guanosine | -0,411817454 | 8,07E-04 |
| iPVR | Guanosine | -0,427174775 | 5,13E-04 |
| iRVESV | Guanosine | -0,479963017 | 6,88E-05 |
| MPAP | Guanosine | -0,480189561 | 6,82E-05 |
| RVEF | Guanosine | 0,441159128 | 2,64E-04 |
| iRV mass | Guanosine | -0,368660193 | 2,59E-03 |
| RVEF | HexCer 40:2;O2 | -0,486692545 | 5,36E-05 |
| ECV RV AIP | HexCer 42:2;O2 | -0,44003123 | 4,35E-04 |
| RVEF | HexCer 42:2;O2 | -0,34987651 | 3,55E-03 |
| ECV RV AIP | Histidine | -0,335365079 | 6,59E-03 |
| ECV RV IIP | Histidine | -0,619491933 | 2,95E-07 |
| ECV SIV | Histidine | -0,320442794 | 8,03E-03 |
| iPVR | Histidine | -0,463826853 | 1,59E-04 |
| iRVESV | Histidine | -0,513582497 | 1,87E-05 |
| MPAP | Histidine | -0,521804176 | 1,33E-05 |
| RVEF | Histidine | 0,671531587 | 3,96E-09 |
| iRV mass | Histidine | -0,451367081 | 2,40E-04 |
| ECV SIV | HP | 0,537428814 | 9,73E-06 |
| ECV RV IIP | Hypotaurine | -0,346722346 | 5,11E-03 |
| iPVR | Hypotaurine | -0,374970449 | 2,20E-03 |
| iRVESV | Hypotaurine | -0,494255677 | 4,02E-05 |
| MPAP | Hypotaurine | -0,410669717 | 6,82E-04 |
| RVEF | Hypotaurine | 0,631583897 | 5,30E-08 |
| iRV mass | Hypotaurine | -0,347200197 | 4,37E-03 |
| ECV RV IIP | Hypoxanthine | -0,544641822 | 1,03E-05 |
| ECV SIV | Hypoxanthine | -0,425499962 | 5,39E-04 |
| iPVR | Hypoxanthine | -0,514924803 | 2,45E-05 |
| iRVESV | Hypoxanthine | -0,543266516 | 5,25E-06 |
| MPAP | Hypoxanthine | -0,482253319 | 6,32E-05 |
| RVEF | Hypoxanthine | 0,488631792 | 4,98E-05 |
| iRV mass | Hypoxanthine | -0,484137953 | 7,82E-05 |
| iRV mass | Inosine | -0,294060532 | 1,39E-02 |
| iRV mass | Isoleucine | 0,47735191 | 9,96E-05 |
| iRV mass | KNG1 | -0,273541995 | 1,89E-02 |
| ECV RV AIP | L-3-Phenyllactic acid | -0,352793472 | 4,44E-03 |
| ECV RV IIP | L-3-Phenyllactic acid | -0,465630064 | 1,94E-04 |
| ECV SIV | L-3-Phenyllactic acid | -0,546111472 | 6,70E-06 |
| iPVR | L-3-Phenyllactic acid | -0,340289563 | 5,14E-03 |
| iRVESV | L-3-Phenyllactic acid | -0,348773706 | 3,65E-03 |
| MPAP | L-3-Phenyllactic acid | -0,55600338 | 2,93E-06 |
| RVEF | L-3-Phenyllactic acid | 0,525488 | 1,14E-05 |
| iRV mass | L-3-Phenyllactic acid | -0,247663626 | 0,032859917 |
| ECV RV IIP | L-Arginine | -0,361396598 | 3,63E-03 |
| RVEF | L-Arginine | 0,320794521 | 7,04E-03 |
| iRV mass | Leucylproline | -0,226446662 | 4,67E-02 |
| ECV RV AIP | L-gamma-Glutamyl-beta-phenyl-beta-L-alanine | 0,41269097 | 9,64E-04 |
| iRVESV | L-gamma-Glutamyl-beta-phenyl-beta-L-alanine | 0,442465155 | 2,53E-04 |
| MPAP | L-gamma-Glutamyl-beta-phenyl-beta-L-alanine | 0,436632265 | 3,06E-04 |
| RVEF | L-gamma-Glutamyl-beta-phenyl-beta-L-alanine | -0,311576723 | 8,64E-03 |
| iRV mass | L-gamma-Glutamyl-beta-phenyl-beta-L-alanine | 0,57585698 | 1,71E-06 |
| iRV mass | L-Homoserine | -0,488361071 | 6,72E-05 |
| iRV mass | Lithocholic acid glycine conjugate | -0,23524291 | 4,05E-02 |
| ECV RV IIP | LPC 17:0/0:0 | 0,50503932 | 4,92E-05 |
| RVEF | LPC(14:0) | 0,59264594 | 4,76E-07 |
| ECV RV AIP | LPC(14:0) | -0,50201783 | 5,49E-05 |
| ECV RV IIP | LPC(14:0) | -0,550533612 | 8,05E-06 |
| ECV SIV | LPC(14:0) | -0,526057074 | 1,56E-05 |
| iPVR | LPC(14:0) | -0,575370975 | 1,75E-06 |
| iRVESV | LPC(14:0) | -0,561293802 | 2,29E-06 |
| MPAP | LPC(14:0) | -0,694357252 | 7,48E-10 |
| iRV mass | LPC(14:0) | -0,49927982 | 4,48E-05 |
| ECV RV IIP | LPC(17:0) | 0,449990064 | 3,20E-04 |
| RVEF | LPC(17:0) | -0,504300897 | 2,72E-05 |
| ECV RV IIP | L-Phenylalanine | -0,373435161 | 2,71E-03 |
| iPVR | L-Phenylalanine | -0,613193257 | 2,53E-07 |
| iRVESV | L-Phenylalanine | -0,330678146 | 5,62E-03 |
| MPAP | L-Phenylalanine | -0,480661454 | 6,70E-05 |
| RVEF | L-Phenylalanine | 0,642262389 | 2,75E-08 |
| iRV mass | L-Pyroglutamic acid | -0,234007485 | 4,13E-02 |
| ECV RV IIP | L-Threonine | -0,616978768 | 3,38E-07 |
| iPVR | L-Threonine | -0,405692362 | 9,61E-04 |
| iRVESV | L-Threonine | -0,321199083 | 6,98E-03 |
| MPAP | L-Threonine | -0,422740455 | 4,73E-04 |
| RVEF | L-Threonine | 0,643545957 | 2,54E-08 |
| iRV mass | L-Threonine | -0,2484545 | 3,24E-02 |
| RVEF | L-Tryptophan | 0,556242224 | 2,90E-06 |
| iRVESV | L-Tryptophan | -0,331764416 | 5,48E-03 |
| MPAP | L-Tryptophan | -0,489397446 | 4,84E-05 |
| ECV RV IIP | L-Tryptophan | -0,565735194 | 4,14E-06 |
| iPVR | L-Tryptophan | -0,653865297 | 2,33E-08 |
| iRV mass | L-Tryptophan | -0,237285728 | 3,91E-02 |
| ECV RV IIP | L-Tyrosine | -0,59753287 | 9,20E-07 |
| ECV SIV | L-Tyrosine | -0,396631099 | 1,24E-03 |
| iPVR | L-Tyrosine | -0,547941967 | 6,18E-06 |
| iRVESV | L-Tyrosine | -0,33044276 | 5,65E-03 |
| MPAP | L-Tyrosine | -0,532592296 | 8,41E-06 |
| RVEF | L-Tyrosine | 0,571495062 | 1,40E-06 |
| iRV mass | Methyluridine | -0,377631553 | 2,06E-03 |
| ECV RV IIP | N6N6-Dimethyladenosine | -0,368981503 | 3,02E-03 |
| iPVR | N6N6-Dimethyladenosine | -0,354388987 | 3,68E-03 |
| iRV mass | PA O-37:0 | 0,243501307 | 3,53E-02 |
| RVEF | PC 16:0/18:2 | -0,348462898 | 3,67E-03 |
| RVEF | PC 18:0/18:1 | -0,432834218 | 3,45E-04 |
| RVEF | PC 18:0/20:3 | -0,42506757 | 4,40E-04 |
| ECV RV IIP | PC 18:0/20:4 | 0,357581387 | 3,97E-03 |
| RVEF | PC 18:0/20:4 | -0,558090003 | 2,66E-06 |
| ECV RV IIP | PC 18:1/18:2 | 0,324083525 | 8,41E-03 |
| RVEF | PC 18:1/18:2 | -0,421482702 | 4,92E-04 |
| RVEF | PC 40:3 | -0,42425714 | 4,52E-04 |
| RVEF | PC O-34:3 | -0,305621872 | 9,82E-03 |
| RVEF | PC O-36:3 | -0,354016585 | 3,20E-03 |
| ECV RV AIP | PC(18:3(9Z12Z15Z)/18:4(6Z9Z12Z15Z)) | 0,547932661 | 8,99E-06 |
| ECV SIV | PC(18:3(9Z12Z15Z)/18:4(6Z9Z12Z15Z)) | 0,52909754 | 1,38E-05 |
| MPAP | PC(18:3(9Z12Z15Z)/18:4(6Z9Z12Z15Z)) | 0,348413467 | 3,68E-03 |
| iRV mass | PC(18:3(9Z12Z15Z)/18:4(6Z9Z12Z15Z)) | 0,351298751 | 3,97E-03 |
| iPVR | PC(20:3(5Z8Z11Z)/20:1(11Z)) | 0,326008689 | 7,10E-03 |
| MPAP | PC(20:3(5Z8Z11Z)/20:1(11Z)) | 0,365595151 | 2,39E-03 |
| RVEF | PC(20:3(5Z8Z11Z)/20:1(11Z)) | -0,482202933 | 6,33E-05 |
| ECV RV AIP | PC(22:1(13Z)/15:0) | 0,367405875 | 3,14E-03 |
| ECV RV IIP | PC(22:1(13Z)/15:0) | 0,518502119 | 2,96E-05 |
| ECV SIV | PC(22:1(13Z)/15:0) | 0,435727043 | 3,95E-04 |
| iPVR | PC(22:1(13Z)/15:0) | 0,496227781 | 5,02E-05 |
| iRVESV | PC(22:1(13Z)/15:0) | 0,378385534 | 1,70E-03 |
| MPAP | PC(22:1(13Z)/15:0) | 0,539719811 | 6,15E-06 |
| RVEF | PC(22:1(13Z)/15:0) | -0,530660203 | 9,14E-06 |
| iRV mass | PC(22:1(13Z)/15:0) | 0,346500836 | 4,45E-03 |
| ECV SIV | PLG | 0,347256076 | 4,37E-03 |
| iRV mass | Proline | -0,25328635 | 0,029815478 |
| iRV mass | Proline betaine | -0,241783308 | 3,63E-02 |
| RVEF | SERPINA1 | -0,499015903 | 3,34E-05 |
| iRVESV | SERPINA6 | 0,330621927 | 5,62E-03 |
| RVEF | SERPINA6 | -0,467056359 | 1,10E-04 |
| RVEF | SERPINA7 | -0,455557811 | 1,63E-04 |
| iRVESV | SERPINC1 | 0,376525467 | 1,79E-03 |
| RVEF | SERPINC1 | -0,535529948 | 7,40E-06 |
| RVEF | SERPIND1 | -0,357195103 | 2,96E-03 |
| iRV mass | SERPIND1 | -0,238080965 | 3,60E-02 |
| iRV mass | SERPINF1 | -0,25558709 | 2,64E-02 |
| RVEF | SERPINF2 | -0,388916623 | 1,28E-03 |
| ECV RV AIP | SM 39:1;O2 | -0,380911675 | 2,24E-03 |
| RVEF | SM 39:1;O2 | -0,387958221 | 1,31E-03 |
| RVEF | SM 41:1;O2 | -0,367933581 | 2,24E-03 |
| ECV RV AIP | SM 43:1;O2 | -0,47473267 | 1,43E-04 |
| RVEF | SM 43:1;O2 | -0,342833212 | 4,21E-03 |
| ECV RV AIP | SM d32:1 | -0,40628979 | 1,15E-03 |
| RVEF | SM d32:1 | -0,361413064 | 2,66E-03 |
| ECV RV AIP | SM d34:1 | -0,343851715 | 5,45E-03 |
| RVEF | SM d34:1 | -0,41879289 | 5,34E-04 |
| ECV RV AIP | SM d35:1 | -0,359040588 | 3,84E-03 |
| RVEF | SM d35:1 | -0,435911799 | 3,13E-04 |
| RVEF | SM d38:2 | -0,482451549 | 6,27E-05 |
| RVEF | SM d40:2 | -0,43485655 | 3,23E-04 |
| ECV RV AIP | SM d41:1 | -0,343627876 | 5,48E-03 |
| RVEF | SM d41:1 | -0,363510463 | 2,52E-03 |
| ECV RV AIP | SM d42:1 | -0,358886101 | 3,85E-03 |
| RVEF | SM d42:1 | -0,373967436 | 1,92E-03 |
| ECV RV AIP | SM(d16:1/18:1) | -0,584347701 | 1,75E-06 |
| ECV RV IIP | SM(d16:1/18:1) | -0,380488869 | 2,27E-03 |
| ECV SIV | SM(d16:1/18:1) | -0,654788628 | 2,19E-08 |
| iPVR | SM(d16:1/18:1) | -0,601529668 | 4,72E-07 |
| iRVESV | SM(d16:1/18:1) | -0,474760179 | 8,31E-05 |
| MPAP | SM(d16:1/18:1) | -0,612793563 | 1,59E-07 |
| iRV mass | SM(d16:1/18:1) | -0,410963399 | 8,27E-04 |
| ECV RV IIP | Dimethylarginine | -0,665146917 | 2,05E-08 |
| ECV SIV | Dimethylarginine | -0,375743453 | 2,16E-03 |
| iPVR | Dimethylarginine | -0,525360408 | 1,61E-05 |
| iRVESV | Dimethylarginine | -0,470133879 | 9,82E-05 |
| MPAP | Dimethylarginine | -0,511101862 | 2,07E-05 |
| RVEF | Dimethylarginine | 0,606288288 | 2,28E-07 |
| iRV mass | Dimethylarginine | -0,437513043 | 3,73E-04 |
| ECV RV IIP | Taurine | -0,39665513 | 1,49E-03 |
| iPVR | Taurine | -0,514446031 | 2,50E-05 |
| iRVESV | Taurine | -0,356337645 | 3,02E-03 |
| MPAP | Taurine | -0,392452301 | 1,16E-03 |
| RVEF | Taurine | 0,610136798 | 1,84E-07 |
| iRV mass | Taurine | -0,256689949 | 2,81E-02 |
| ECV RV IIP | TG 55:9/PC44:5 | 0,372355262 | 2,78E-03 |
| RVEF | TG 55:9/PC44:5 | -0,579223436 | 9,51E-07 |
| MPAP | TG 56:2 | -0,31461906 | 8,08E-03 |
| RVEF | TG 58:10 | -0,330605644 | 5,63E-03 |
| MPAP | TG 58:3 | -0,319661226 | 7,22E-03 |
| iRV mass | TG 58:3 | -0,245956408 | 3,38E-02 |
| RVEF | TG 58:9 | -0,319999341 | 7,17E-03 |
| RVEF | TG 60:7 | -0,320512255 | 7,09E-03 |
| iPVR | TG 62:6 | -0,334786186 | 5,83E-03 |
| MPAP | TG 62:6 | -0,366811238 | 2,31E-03 |
| iRV mass | TG 62:6 | -0,230119103 | 4,40E-02 |
| MPAP | TG 62:7 | -0,327519295 | 6,04E-03 |
| iRV mass | TG 62:7 | -0,287215567 | 1,59E-02 |
| ECV RV IIP | TG(60:5) | 0,335882659 | 6,51E-03 |
| RVEF | TG(60:5) | -0,327889551 | 5,99E-03 |
| ECV RV IIP | Threonine | -0,551376431 | 7,76E-06 |
| ECV SIV | Threonine | -0,399610546 | 1,14E-03 |
| iPVR | Threonine | -0,31058783 | 9,91E-03 |
| MPAP | Threonine | -0,354171428 | 3,19E-03 |
| RVEF | Threonine | 0,425835474 | 4,30E-04 |
| ECV RV IIP | Tyrosine | -0,543346195 | 1,09E-05 |
| ECV SIV | Tyrosine | -0,437314719 | 3,76E-04 |
| iPVR | Tyrosine | -0,485381616 | 7,48E-05 |
| iRVESV | Tyrosine | -0,372820515 | 1,97E-03 |
| MPAP | Tyrosine | -0,437279759 | 2,99E-04 |
| RVEF | Tyrosine | 0,4609033 | 1,36E-04 |
| iRV mass | Tyrosine | -0,231371184 | 4,31E-02 |
| ECV RV IIP | Xanthine | -0,691187877 | 3,61E-09 |
| iPVR | Xanthine | -0,504713563 | 3,64E-05 |
| MPAP | Xanthine | -0,427067127 | 4,14E-04 |
| RVEF | Xanthine | 0,561803733 | 2,23E-06 |
| iRV mass | Xanthine | -0,242019662 | 3,62E-02 |
| ECV RV IIP | Xanthosine | -0,498878851 | 6,16E-05 |
| iPVR | Xanthosine | -0,409989342 | 8,50E-04 |
| MPAP | Xanthosine | -0,418237347 | 5,43E-04 |
| RVEF | Xanthosine | 0,448053249 | 2,10E-04 |

**Supplementary Table 11.** Association between the main identified metabolites and RV ejection fraction adjusted by weight.

|  | B coefficient | 95% CI | P value | P value for weight as covariate |
| --- | --- | --- | --- | --- |
| FA 20:3 | -4.203 | -8.164, -0.243 | 0.038 | 0.111 |
| FA 22:4 | -3.434 | -6.721, -0.147 | 0.041 | 0.056 |
| FA 18:2 | -2.158 | -4.291, -0.026 | 0.047 | 0.078 |
| FA 20:2 | -2.684 | -5.161, -0.208 | 0.035 | 0.073 |
| FA 20:4 | -3.399 | -6.196, -0.601 | 0.019 | 0.076 |
| Histidine | 5.200 | 2.429, 7.970 | 0.001 | 0.205 |
| Arginine | 6.475 | 1.599, 11,352 | 0.011 | 0.154 |
| Guanine | 5.189 | 2.043, 8.335 | 0.002 | 0.058 |
| Hypotaurine | 4,632 | 1,132, 9,132 | 0.044 | 0.171 |
| Guanosine | 2.874 | 0.084, 5.665 | 0.044 | 0.185 |

**4. SUPPLEMENTARY FIGURES**

**Supplementary Figure 1:** Programmed surgeries to achieve the final number of experimental animals in the study.

**
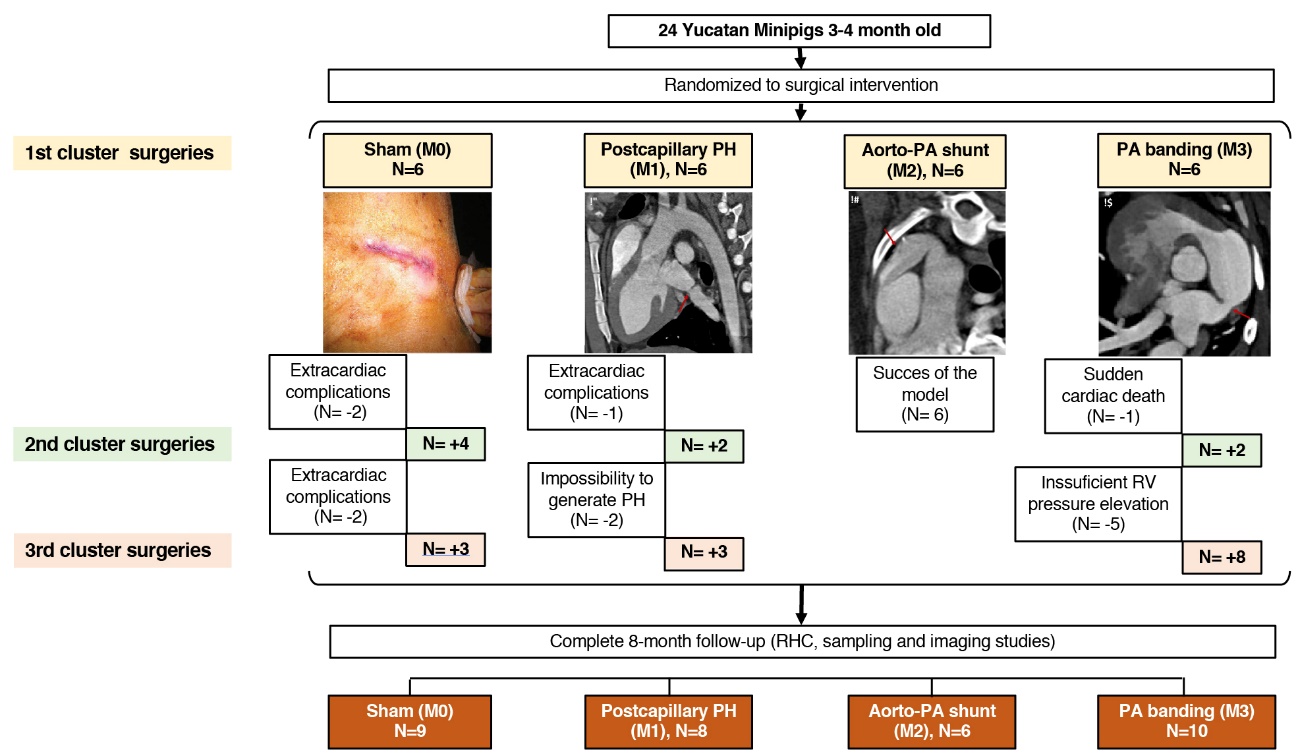
**

PA: Pulmonary artery; PH: Pulmonary hypertension; RV: Right ventricular.

**Supplementary Figure 2:** Serum content of complement system and coagulation cascade proteins

Volcano plots show fold change values (Log2, x-axis) against the adjusted p-value (-Log10 of p-value, y-axis) in the concentration of coagulation and complement system cascade components Significantly downregulated biomarkers are highlighted red, and significantly upregulated biomarkers are highlighted green; p<0.05. Changes in protein expression were determined by multiplexed immunoassays in plasma samples of non-oxygenated blood **(A)** or oxygenated blood **(B)**. Data are expressed as the fold change relative to M0 samples. Differences were analyzed by multiple t-tests with false discovery rate (FDR) correction using the Benjamini and Hochberg method.

**Supplementary Figure 3:** CILP1 and NT-proBNP levels in the 3 experimental models and the sham-operated group.


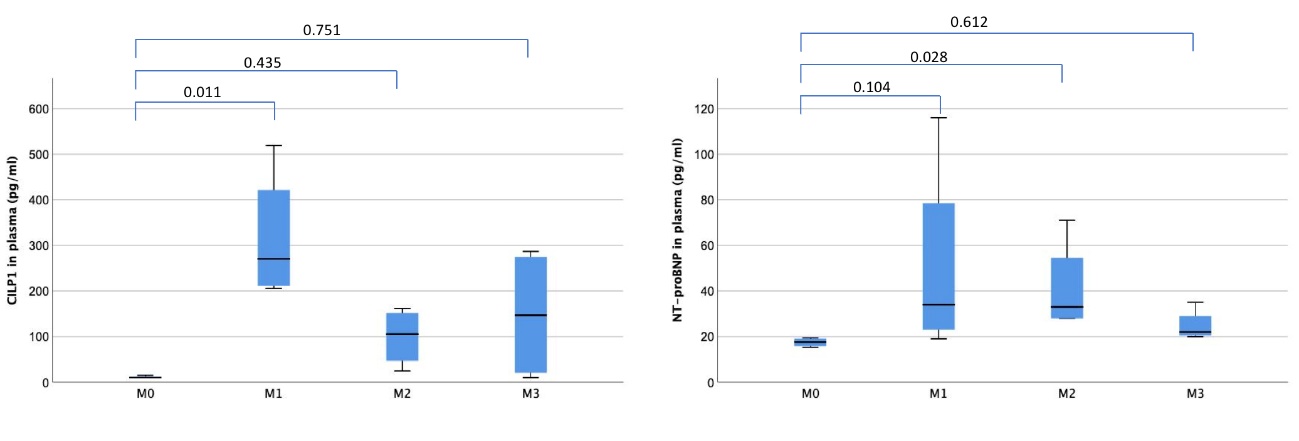


The box and whisker plot shows the median values and variation of plasmatic CILP1 and NT-proBNP levels at end of follow-up in the three disease models (M1-M3) compared to Sham-operated group (M0). The comparison was performed by Mann-Whitney test with p value correction for multiple comparisons. P-vales are shown on top of each comparative bar.

**Supplementary Figure 4:** Gene expression changes in cardiac and pulmonary tissues


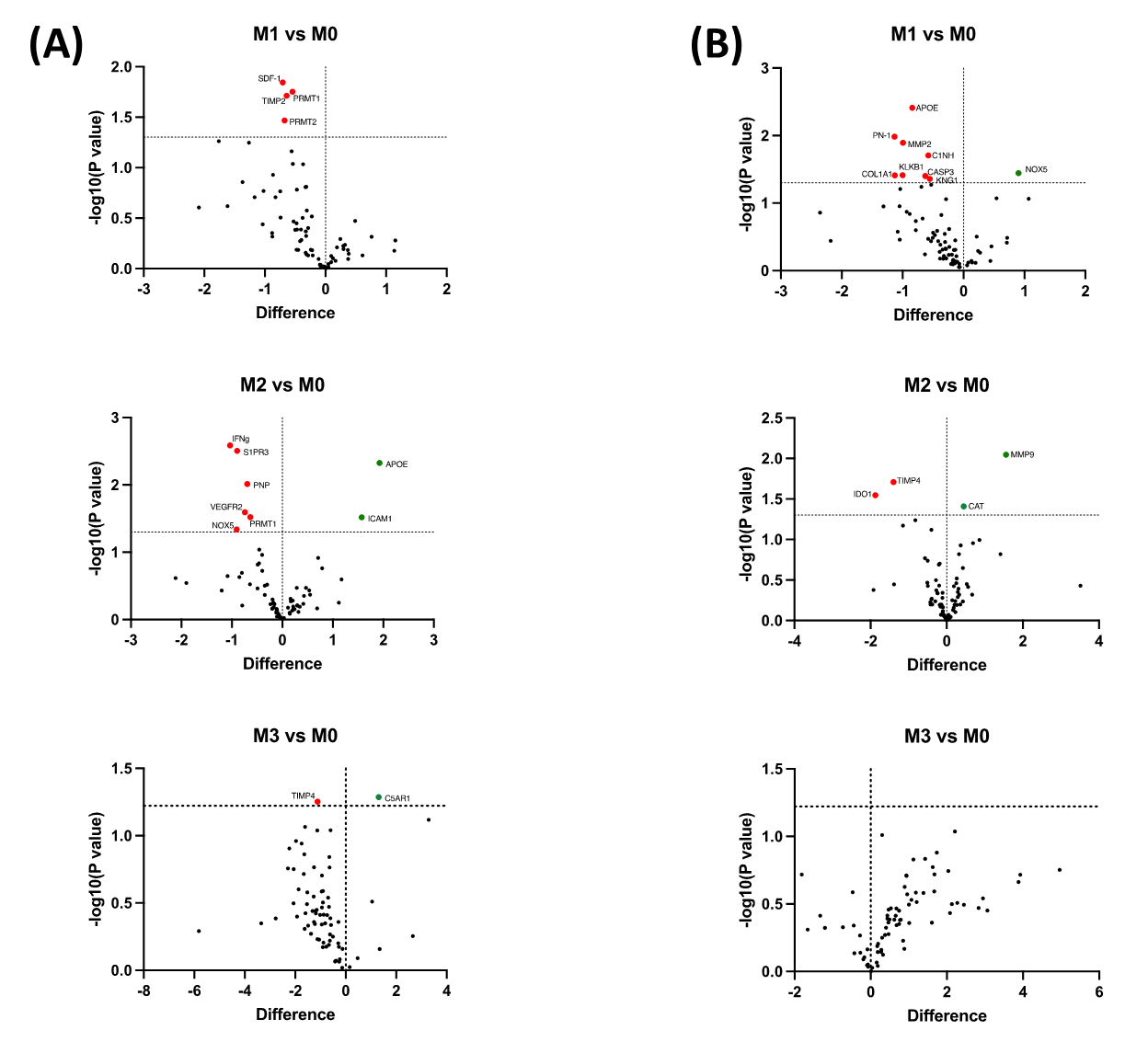


Volcano plots show fold change values (Log2, x-axis) against the adjusted p-value (-Log10 of p-value, y-axis) in mRNA expression. Significantly downregulated biomarkers are highlighted red, and significantly upregulated biomarkers are highlighted green; p<0.05. Changes in gene expression were determined by quantitative PCR in RV myocardium **(A)** or lung parenchyma **(B)**. Data are expressed as the fold change relative to M0 samples. Differences were analyzed by multiple t-tests with false discovery rate (FDR) correction using the Benjamini and Hochberg method.

**5. SUPPLEMENTARY REFERENCES**

1. Garcia-Alvarez A, Fernandez-Friera L, Garcia-Ruiz JM, Nuno-Ayala M, Pereda D, Fernandez-Jimenez R, Guzman G, Sanchez-Quintana D, Alberich-Bayarri A, Pastor-Escuredo D, Sanz-Rosa D, Garcia-Prieto J, Gonzalez-Mirelis JG, Pizarro G, Jimenez-Borreguero LJ, Fuster V, Sanz J, Ibanez B. Noninvasive monitoring of serial changes in pulmonary vascular resistance and acute vasodilator testing using cardiac magnetic resonance. J Am Coll Cardiol. 2013;62(17):1621-31.

2. Pereda D, Garcia-Alvarez A, Sanchez-Quintana D, Nuno M, Fernandez-Friera L, Fernandez-Jimenez R, Garcia-Ruiz JM, Sandoval E, Aguero J, Castella M, Hajjar RJ, Fuster V, Ibanez B. Swine model of chronic postcapillary pulmonary hypertension with right ventricular remodeling: long-term characterization by cardiac catheterization, magnetic resonance, and pathology. J Cardiovasc Transl Res. 2014;7(5):494-506.

3. Garcia-Alvarez A, Garcia-Lunar I, Pereda D, Fernandez-Jimenez R, Sanchez-Gonzalez J, Mirelis JG, Nuno-Ayala M, Sanchez-Quintana D, Fernandez-Friera L, Garcia-Ruiz JM, Pizarro G, Aguero J, Campelos P, Castella M, Sabate M, Fuster V, Sanz J, Ibanez B. Association of myocardial T1-mapping CMR with hemodynamics and RV performance in pulmonary hypertension. JACC Cardiovasc Imaging. 2015;8(1):76-82.

4. Pereda D, Garcia-Lunar I, Sierra F, Sanchez-Quintana D, Santiago E, Ballesteros C, Encalada JF, Sanchez-Gonzalez J, Fuster V, Ibanez B, Garcia-Alvarez A. Magnetic Resonance Characterization of Cardiac Adaptation and Myocardial Fibrosis in Pulmonary Hypertension Secondary to Systemic-To-Pulmonary Shunt. Circulation Cardiovasc Imaging. 2016;9(9).

5. Garcia-Lunar I, Pereda D, Santiago E, Solanes N, Nuche J, Ascaso M, Bobi J, Sierra F, Dantas AP, Galan C, San Antonio R, Sanchez-Quintana D, Sanchez-Gonzalez J, Barbera JA, Rigol M, Fuster V, Ibanez B, Sabate M, Garcia-Alvarez A. Effect of pulmonary artery denervation in postcapillary pulmonary hypertension: results of a randomized controlled translational study. Basic Res Cardiol. 2019;114(2):5.

6. Garcia-Lunar I, Blanco I, Fernandez-Friera L, Prat-Gonzalez S, Jorda P, Sanchez J, Pereda D, Pujadas S, Rivas M, Sole-Gonzalez E, Vazquez J, Blazquez Z, Garcia-Picart J, Caravaca P, Escalera N, Garcia-Pavia P, Delgado J, Segovia-Cubero J, Fuster V, Roig E, Barbera JA, Ibanez B, Garcia-Alvarez A. Design of the beta3-Adrenergic Agonist Treatment in Chronic Pulmonary Hypertension Secondary to Heart Failure Trial. JACC Basic Transl Sci. 2020;5(4):317-27.

7. Garcia-Alvarez A, Blanco I, Garcia-Lunar I, Jorda P, Rodriguez-Arias JJ, Fernandez-Friera L, Zegri I, Nuche J, Gomez-Bueno M, Prat S, Pujadas S, Sole-Gonzalez E, Garcia-Cossio MD, Rivas M, Torrecilla E, Pereda D, Sanchez J, Garcia-Pavia P, Segovia-Cubero J, Delgado JF, Mirabet S, Fuster V, Barbera JA, Ibanez B, Investigators S-H. beta3 adrenergic agonist treatment in chronic pulmonary hypertension associated with heart failure (SPHERE-HF): a double blind, placebo-controlled, randomized clinical trial. Eur J Heart Fail. 2023;25(3):373-85.

8. Carnicelli AP, Stone JJ, Doyle A, Chowdhry AK, Mix D, Ellis J, Gillespie DL, Chandra A. Cross-sectional area for the calculation of carotid artery stenosis on computed tomographic angiography. J Vasc Surg. 2013;58(3):659-65.

9. Sartain, M.; Salcedo, J.; Murali, A.; Li, X.; Stow, S.; Koelmel, J. Improving Coverage of the Plasma Lipidome Using Iterative MS/MS Data Acquisition Combined with Lipid Annotator Software and 6546 LC/Q-TOF. Agilent Application Note 2019, 5994–0775en. Available online: https://www.agilent.com/cs/library/applications/application-6546-q-tof-lipidome-5994-0775en-agilent.pdf (accessed on 25 March 2022).

10. Pellegrino RM, Di Veroli A, Valeri A, Goracci L, Cruciani G. LC/MS lipid profiling from human serum: a new method for global lipid extraction. Anal Bioanal Chem. 2014;406(30):7937-48.

11. Koelmel JP, Li X, Stow SM, Sartain MJ, Murali A, Kemperman R, Tsugawa H, Takahashi M, Vasiliou V, Bowden JA, Yost RA, Garrett TJ, Kitagawa N. Lipid Annotator: Towards Accurate Annotation in Non-Targeted Liquid Chromatography High-Resolution Tandem Mass Spectrometry (LC-HRMS/MS) Lipidomics Using A Rapid and User-Friendly Software. Metabolites. 2020;10(3).

12. Moran-Garrido M, Munoz-Escudero P, Garcia-Alvarez A, Garcia-Lunar I, Barbas C, Saiz J. Optimization of sample extraction and injection-related parameters in HILIC performance for polar metabolite analysis. Application to the study of a model of pulmonary hypertension. J Chromatogr A. 2022;1685:463626.

13. Gil-de-la-Fuente A, Godzien J, Saugar S, Garcia-Carmona R, Badran H, Wishart DS, Barbas C, Otero A. CEU Mass Mediator 3.0: A Metabolite Annotation Tool. J Proteome Res. 2019;18(2):797-802.

14. Martinez-Bartolome S, Navarro P, Martin-Maroto F, Lopez-Ferrer D, Ramos-Fernandez A, Villar M, Garcia-Ruiz JP, Vazquez J. Properties of average score distributions of SEQUEST: the probability ratio method. Mol Cell Proteomics. 2008;7(6):1135-45.

15. Navarro P, Vazquez J. A refined method to calculate false discovery rates for peptide identification using decoy databases. J Proteome Res. 2009;8(4):1792-6.

16. Trevisan-Herraz M, Bagwan N, Garcia-Marques F, Rodriguez JM, Jorge I, Ezkurdia I, Bonzon-Kulichenko E, Vazquez J. SanXoT: a modular and versatile package for the quantitative analysis of high-throughput proteomics experiments. Bioinformatics. 2019;35(9):1594-6.

17. Navarro P, Trevisan-Herraz M, Bonzon-Kulichenko E, Nunez E, Martinez-Acedo P, Perez-Hernandez D, Jorge I, Mesa R, Calvo E, Carrascal M, Hernaez ML, Garcia F, Barcena JA, Ashman K, Abian J, Gil C, Redondo JM, Vazquez J. General statistical framework for quantitative proteomics by stable isotope labeling. J Proteome Res. 2014;13(3):1234-47.

18. Garcia-Marques F, Trevisan-Herraz M, Martinez-Martinez S, Camafeita E, Jorge I, Lopez JA, Mendez-Barbero N, Mendez-Ferrer S, Del Pozo MA, Ibanez B, Andres V, Sanchez-Madrid F, Redondo JM, Bonzon-Kulichenko E, Vazquez J. A Novel Systems-Biology Algorithm for the Analysis of Coordinated Protein Responses Using Quantitative Proteomics. Mol Cell Proteomics. 2016;15(5):1740-60.

19. Szklarczyk D, Gable AL, Nastou KC, Lyon D, Kirsch R, Pyysalo S, Doncheva NT, Legeay M, Fang T, Bork P, Jensen LJ, von Mering C. The STRING database in 2021: customizable protein-protein networks, and functional characterization of user-uploaded gene/measurement sets. Nucleic Acids Res. 2021;49(D1):D605-D12.

20. Perez-Riverol Y, Bai J, Bandla C, Garcia-Seisdedos D, Hewapathirana S, Kamatchinathan S, Kundu DJ, Prakash A, Frericks-Zipper A, Eisenacher M, Walzer M, Wang S, Brazma A, Vizcaino JA. The PRIDE database resources in 2022: a hub for mass spectrometry-based proteomics evidences. Nucleic Acids Res. 2022;50(D1):D543-D52.

21. Sud M, Fahy E, Cotter D, Azam K, Vadivelu I, Burant C, Edison A, Fiehn O, Higashi R, Nair KS, Sumner S, Subramaniam S. Metabolomics Workbench: An international repository for metabolomics data and metadata, metabolite standards, protocols, tutorials and training, and analysis tools. Nucleic Acids Res. 2016;44(D1):D463-70.

22. Palstrom NB, Matthiesen R, Rasmussen LM, Beck HC. Recent Developments in Clinical Plasma Proteomics-Applied to Cardiovascular Research. Biomedicines. 2022;10(1).
